# A Mg‐Chelatase Subunit I Missense Mutant in Barley Exhibits a Cold‐Sensitive Phenotype Under Field Conditions

**DOI:** 10.1111/ppl.70434

**Published:** 2025-08-05

**Authors:** Andrea Persello, Viola Torricella, Federico Ballabio, Chiara Bertaso, Lisa Rotasperti, Nicolaj Jeran, Simona Masiero, Nina Gauri Capra, Riccardo Capelli, Carlo Camilloni, Laura Rossini, David S. Horner, Francesco Camerlengo, Giuseppe Sangiorgi, Anja Krieger‐Liszkay, Silvan Petrac, Luca Tadini, Bernhard Grimm, Paolo Pesaresi

**Affiliations:** ^1^ Department of Biosciences University of Milan Milan Italy; ^2^ Department of Industrial Engineering University of Padua Padua Italy; ^3^ Institute of Biophysics, National Research Council Milano Italy; ^4^ Department of Agricultural and Environmental Sciences ‐ Production, Landscape, Agroenergy University of Milan Milan Italy; ^5^ Department of Agricultural and Food Sciences University of Bologna Bologna Italy; ^6^ Université Paris‐Saclay Institute for Integrative Biology of the Cell (I2BC) Gif‐sur‐Yvette France; ^7^ Institute of Biology/Plant Physiology, Humboldt‐Universität zu Berlin Berlin Germany

**Keywords:** barley, cold‐sensitive phenotype, Mg‐chelatase, pale green mutant, photosynthesis

## Abstract

The pale‐green barley mutant *xan‐h.chli‐1* has a *Hv*CHLI subunit of Mg‐chelatase with an Arg‐to‐Lys substitution at position 298 and exhibits a unique cold‐sensitive phenotype. Under winter field conditions, *xan‐h.chli‐1* plants fail to survive, whereas they thrive under spring or greenhouse conditions. Controlled experiments show a specific cold‐induced chlorosis gradient along leaf blades of the mutant that was not observed in other pale‐green mutants with either altered chlorophyll biosynthesis, such as *chlorina‐f2.101* and *chlorina.111*, or defects in photosystem antenna protein assembly, such as *hus1*. Photosynthetic function in young leaf tissues was restored when exposed to optimal temperatures, emphasizing the reversibility of this cold stress. Molecular dynamics simulations revealed a temperature‐dependent disruption of the interaction of ATP with Lys298 in the *Hv*CHLI subunit, which correlates with the observed cold sensitivity. Transcriptomic analyses revealed distinct gene expression patterns under cold stress in *xan‐h.chli‐1* leaves, which were characterized by marked inactivation of genes related to pigment biosynthesis, photosynthesis, cell‐wall formation, and remodeling. These findings highlight the role of the *xan‐h.chli‐1* mutation in conferring cold sensitivity and offer new insights into molecular strategies for introducing the pale‐green trait into modern crop varieties.

## 1 Introduction

Although plant architecture and grain yield have been extensively studied in modern breeding programs, photosynthetic traits have often been overlooked despite their considerable potential to enhance both crop productivity and resilience to changing climatic conditions (Long et al. 2015). In this context, the solar energy conversion efficiency index (ECE)—which reflects the proportion of absorbed solar radiation that is converted into biomass—is one of the key parameters. Indeed, the total biomass production of crops is closely linked to the ECE, which in turn depends on the photosynthetic efficiency at the canopy level (Slattery and Ort 2020). However, in many crops, ECE remains below 50% of its theoretical maximum (Slattery and Ort 2020). This is because, in natural ecosystems, plants must compete with other species for light, often accumulating high amounts of chlorophyll and thylakoid‐localized antenna proteins that are higher than necessary for optimal photosynthetic performance (Canham et al. 1994). In fact, the photosynthetic apparatus in C3 plant canopies becomes saturated at approximately 25% of the maximum solar flux (Jansson et al. 2010). Furthermore, in cultivated environments such as monocultures, intra‐species competition for nutrients can be detrimental, suggesting that cultivars with reduced chlorophyll content could have productive advantages in these systems. Hence, the reduction of leaf chlorophyll content has been proposed as a strategy for improving canopy light penetration and photosynthetic efficiency in densely planted crops by alleviating photo‐oxidative damage caused by high light intensity and ultimately enhancing biomass production (Melis 2009). In addition, the reduced content of chlorophyll and chlorophyll‐binding proteins is hypothesized to increase the efficiency of nitrogen use in crops (Walker et al. 2017; Sakowska et al. 2018).

In accordance with these ideas, pale‐green crops can be developed by manipulating various biological processes, including the biogenesis and accumulation of antenna proteins—an approach known as truncated light‐harvesting antennae (TLA; Kirst et al. 2017) – as well as pigment biosynthesis (Cutolo et al. 2023). For example, a pale‐green tobacco line with reduced expression of the *cpSRP43* gene exhibited increased photosynthetic efficiency and enhanced biomass accumulation when grown at high density under greenhouse conditions (Kirst et al. 2018). The *cpSRP43* gene encodes the 43 kDa component of the chloroplast signal recognition particle pathway, which is crucial for the post‐translational targeting of light‐harvesting complex proteins to the thylakoid membranes. Conversely, manipulation of the tetrapyrrole biosynthesis pathway can lead to undesirable pleiotropic effects, as seen in the pale‐green soybean mutants *MinnGold* and *Y11y11*, both of which have mutations in the Mg‐chelatase I subunit (ChlI1a). In each of these mutants, the plant growth rate is reduced (Sakowska et al. 2018; Campbell et al. 2015), where *MinnGold* showed a 26% decrease in biomass production under field conditions, most likely due to decreased chlorophyll accumulation causing slower NPQ relaxation after sudden transitions from high to low light intensities. Reduced chlorophyll content may also affect the plant's ability to adapt to environmental stresses, as chloroplasts are major sensors of environmental changes and play a key role in cellular stress responses in plants (Fernández and Strand 2008). Furthermore, mutants with defects in the chlorophyll biosynthesis pathway can be more sensitive to high light, as observed in several wheat mutants ( *Triticum aestivum* L.), characterized by a reduced ability to regulate electron poise during a fast rise in irradiance (Falbel et al. 1996; Ferroni et al. 2020).

In this study, we further characterized the pale‐green barley mutant line *xan‐h.chli‐1*, in which a single point mutation in the *Xan‐h* locus encoding the Mg‐chelatase subunit *Hv*CHLI results in an Arg‐to‐Lys substitution at position 298 (Arg298Lys; Persello et al. 2024). Mg‐chelatase is a multimeric complex (comprised of *Hv*CHLI, *Hv*CHLD, *Hv*CHLH, and the regulatory subunit *Hv*Gun4) that is responsible for inserting Mg^2+^ into protoporphyrin IX and marks the first step in the chlorophyll biosynthesis pathway. Under controlled growth conditions, mutant plants show reduced accumulation of antenna and photosystem core subunits, along with a decrease in photosystem II (PSII) yield under moderate light intensities relative to the wild type, but exhibit consistently higher PSII yield at high light intensities. Moreover, the reduced chlorophyll content in leaves is linked to a stable decrease in the daily transpiration rate, together with slight decreases in total biomass accumulation and water‐use efficiency—traits reminiscent of wild barley accessions and landraces that have adapted to arid climates (Persello et al. 2024). Notably, the *xan‐h.chli‐1* mutant exhibits a cold‐sensitive phenotype under both field and low‐temperature growth conditions, which results in impaired chlorophyll accumulation and altered chloroplast ultrastructure. Here, we provide a detailed characterization of the cold‐sensitive phenotype observed in *xan‐h.chli‐1* mutant plants after prolonged cold exposure and present a model that explains the molecular mechanism underlying this phenotype.

## 2 Materials and Methods

### 2.1 Nucleotide and Amino‐Acid Sequence Analysis

The following genome sequences of *Xan‐h* were obtained from the ENSAMBLE‐Plant database: “MorexV3 pseudomolecules assembly”: *HvCHLI*, (*HORVU.MOREX.r3.7HG0738240*), *HvCHLD* (*HORVU.MOREX.r3.5HG0524250*), *HvCHLH* (*HORVU.MOREX.r3.2HG0117630*), *HvGun4* (*HORVU.MOREX.r3.4HG0343310*), *HvSCA3* (*HORVU.MOREX.r3.7HG0742200*), *HvWRKY40* (*HORVU.MOREX.r3.6HG0568570*), *HvHSFA4A* (*HORVU.MOREX.r3.1HG0082040*), *HvLOX2* (*HORVU.MOREX.r3.7HG0679840*), *HvActin7* (*HORVU.MOREX.r3.1HG0003140*), *HvcpSRP43* (*HORVU.MOREX.r3.4HG0409840*), *Hv‐CAO* (*HORVU.MOREX.r3.3HG0324440*), *HvFLOT3* (*HORVU.MOREX.r3.6HG0567430*), *HvCAT1* (*HORVU.MOREX.r3.6HG0545480*) and *HvCRD1* (*HORVU.MOREX.r3.3HG0257850*; plants.ensembl.org/index.html). The amino‐acid sequences were obtained from the UniProtKB (https://www.uniprot.org/help/uniprotkb) and ENSAMBLE‐Plant databases.

### 2.2 Plant Material

Mutant lines used in this work are listed in Table S1. The *xan‐h.chli‐1/xan‐h.chli‐1* line was obtained by backcrossing *TM2490* (mutated in the *Xan‐h* gene, *HORVU.MOREX.r3.7HG0738240*) with the barley cv. Morex (*BC*_2_ *F*_2_). *TM2490* was identified among the M4 generation of the chemically mutagenized TILLMore population (Talamè et al. 2008) in the cv. Morex background. The *BC*_2_ *F*_2_ segregating population was obtained by manually crossing the *xan‐h.chli‐1/xan‐h.chli‐1* line with the elite cv. RGT‐Planet. The T‐DNA Express database (signal.salk.edu/cgi‐bin/tdnaexpress) was used to identify the *Atchli1/Atchli1* T‐DNA insertion mutant (*SAIL 230 D11*), which is characterized by a seedling‐lethal phenotype (Persello et al. 2024), while the homozygous line *cs/cs* was provided by Professor Tatsuru Masuda (Kobayashi et al. 2008). The transgenic Arabidopsis lines *Atchli1/Atchli1+35S::Xan‐h* and *Atchli1/Atchli1+35S::xan‐h.chli‐1* were generated as previously reported (Persello et al. 2024).

### 2.3 Plant Growth Conditions

Barley ( *Hordeum vulgare* ) plants were cultivated under controlled greenhouse conditions (200 μmol photons m^*−*2^ s^*−*1^ for 16 and 8 h dark) on acid soil (Vigor plant‐growth medium, based on Irish and Baltic peats, pH 6.0) supplemented with Osmocote fertilizer. Temperatures were set to 22°C during the day and 16°C at night, with a relative humidity of 30%. The experimental fields were hosted by the Botanical Garden “Città Studi” at the University of Milan (45°28^*′*^32.2^*′′*^N—9°14^*′*^05.0^*′′*^E). Seeds were sown at a density of 100 seeds per row in March and November of the years 2022 and 2023. Arabidopsis Columbia‐0 (Col‐0) and mutant lines were grown on soil (acid sphagnum peat, Atami Bio‐Gromix; pot volume 0.5 L) in a climate chamber (Percival CLF AR‐66L; 150 μmol photons m^*−*2^ s^*−*1^ for 16 h, and 8 h dark, 22°C and a relative humidity of 60%) in the Department of Biosciences at the University of Milan (45°28*′*32.2*′′*N—9°14*′*05.0*′′*E).

### 2.4 Barley and Arabidopsis Cold Stress and Recovery

Cold stress was induced in barley by growing seedlings for the first 14 days under optimal greenhouse conditions, followed by 25 days at 4°C under 100 μmol photons m^*−*2^ s^*−*1^ for 16 h light and 8 h dark. Recovery from cold stress was induced by returning the cold‐adapted plants to optimal greenhouse conditions for 5 days. Arabidopsis seedlings were grown for 16 days under controlled climate‐chamber conditions (22°C) and then exposed to cold stress for 50 days at 4°C. Recovery was induced by transferring the cold‐adapted Arabidopsis plants back to 22°C under growth‐chamber conditions for 5 days.

### 2.5 Barley High‐Light Stress and Recovery

High‐light conditions were obtained by growing barley plants for 14 days at 1000 μmol photons m^*−*2^ s^*−*1^ and control plants were grown in low light at 150 μmol photons m^*−*2^ s^*−*1^. Recovery was induced by transferring plants from high to low light levels for 5 days.

### 2.6 Immunoblot Analysis

Thylakoids and total protein extracts were prepared from equal amounts of barley leaves (fresh weight) as described previously (Bassi and Simpson 1987). Protein extracts were fractionated on denaturing 12% (w/v) polyacrylamide gels in the presence of Tris‐glycine SDS (Schägger and von Jagow 1987) and transferred to polyvinylidene‐difluoride (PVDF) membranes (Ihnatowicz et al. 2004). Signals were detected by enhanced chemiluminescence (GE Healthcare).

*Hv*Lhca1 (AS01 005), *Hv*Lhcb1 (AS01 004), *Hv*PsaD (AS09461), *Hv*PsbO (AS05092), *Hv*H3 (AS10710) and *Hv*β *−* ATP (AS164058) were obtained from Agrisera. Antibodies were raised against *Hv*CHLI, *Hv*CHLD, and *Hv*CHLH as previously described (Lake et al. 2004). The *Hv*Gun4‐specific antibody was kindly provided by Professor Mats Hansson (Lund University, Sweden). For relative quantification, 100%, 50%, and 25% dilutions of *Xan‐h* or Col‐0 protein extracts were loaded. One filter representative of three biological replicates is shown for each immunoblot. Protein extracts were normalized based on leaf fresh weight.

### 2.7 Analysis of Chlorophyll, Tetrapyrrole Precursors, and 5‐Aminolevulinic Acid

Total leaf chlorophyll content was measured in vivo using the SPAD‐502 chlorophyll meter (Konica‐Minolta) on a minimum of six plants for each genotype and condition. Leaf material was harvested, weighed (25–30 mg), and homogenized in liquid nitrogen using a Ball Mill (Retsch). Chlorophyll *a*, Chlorophyll *b*, and tetrapyrrole intermediates were extracted in frozen leaf powder using pre‐cooled basic acetone (acetone: 0.2 M NH_4_OH; 9:1, v/v). Samples were incubated for 20 min at −20°C and centrifuged for 30 min (4°C, 16100 ×g). After centrifugation (12,000 ×g, 15 min, and 4°C) the clear supernatant was analyzed by high‐performance liquid chromatography (HPLC) using the 1260 Infinity II Prime LC system (Agilent) HPLC as previously described in Czarnecki and Grimm (2012) with small modifications.

For analysis of the 5‐aminolevulinic acid (ALA), leaves were cut in the lower, middle, and upper parts, placed vertically into a small beaker filled with 50 mM Tris–HCl buffer (pH 7.2) containing 40 mM levulinic acid, and incubated for 4 h in the light (120 μmol photons m^−2^ s^−1^; 22°C) under constant airflow. The ALA content was determined as previously described (Mauzerall and Granick 1956) and normalized to fresh weight.

### 2.8 Chlorophyll Fluorescence Measurements

An imaging chlorophyll fluorometer (Imaging PAM; Walz) was used for the in vivo imaging of Chl *a* fluorescence on four independent leaves for each genotype/condition. Dark‐adapted plants were exposed to the blue measuring beam (1 Hz, intensity 4) and a saturating light flash (intensity 10) was used to determine the maximum quantum photosynthetic yield (Fv/Fm) values. A 5‐min exposure to actinic light (56 μmol photons m^*−*2^ s^*−*1^ for *A. thaliana* plants and 90 μmol photons m^*−*2^ s^*−*1^ for barley leaves) was applied to calculate the effective quantum yields of PSII (Y[II]). The Handy PEA fluorometer (Hansatech Instruments Ltd.) was used to measure Fv/Fm and Y(II) values in barley plants grown under field conditions.

### 2.9 Spin Trapping ROS Detection

Intact chloroplasts were isolated from the second leaf of *Xan‐h* and *xan‐h.chli‐1* plants grown for the first 14 days under optimal greenhouse conditions, followed by 10 days at 4°C under 100 μmol photons m^*−*2^ s^*−*1^ for 16 light and 8 h dark. Chloroplasts were isolated through blending in 0.33 M sorbitol, 1 mM EDTA, 1 mM MgCl_2_, 50 mM KCl, and 25 mM MES pH = 6.1, followed by washing in 0.33 M sorbitol, 1 mM EDTA, 1 mM MgCl_2_, 50 mM KCl, and 25 mM HEPES pH = 6.7, and resuspension in 0.33 M sorbitol, 1 mM EDTA, 1 mM MgCl_2_, 50 mM KCl, and 25 mM HEPES pH = 7.6.

To detect O_2_^●−^/H_2_O_2_–derived hydroxyl radicals in illuminated thylakoids, the spin trap α‐(4‐pyridyl‐1‐oxide)‐N‐tert‐butylnitron (4‐POBN) was used. The addition of 50 μM Fe‐EDTA generated ^●^OH from H_2_O_2_ via the so‐called Fenton reaction. Ethanol 4% was added to allow the reaction between the spin trap and H_2_O_2_‐derived hydroxyl radicals. Samples were illuminated for 2 min with white light (500 μmol quanta m^−2^ s^−1^) in the presence of 50 mM 4‐POBN, 4% ethanol, 50 μM Fe‐EDTA, and buffer (25 mM HEPES, pH 7.5, 5 mM MgCl_2_, 0.3 M sorbitol).

### 2.10 Transmission Electron Microscopy (TEM)

TEM analyses were performed as described previously (Tadini et al. 2020). Sections (2 × 3 mm) of the apical, middle, and basal second‐leaf sections of *Xan‐h* and *xan‐h.chli‐1* barley plants were manually dissected and fixed under vacuum in 2.5% (w/v) glutaraldehyde and 0.1 M sodium cacodylate buffer. Samples were washed several times with water and counterstained with 0.5% uranyl acetate (w/v) overnight at 4°C. Tissues were then dehydrated in increasing concentrations of ethanol (70%, 80%, 90%, 100% v/v) and permeated twice with 100% (v/v) propylene oxide. The samples were first infiltrated with a 1:2 mixture of Epon‐Araldite and propylene oxide for 2 h, then with Epon‐Araldite and propylene oxide (1:1) for 1 h and left in a 2:1 mixture of Epon‐Araldite and propylene oxide overnight at room temperature. Epon‐Araldite resin was prepared by mixing Embed 812, Araldite 502, dodecenylsuccinic anhydride (DDSA) and Epon Accelerator DMP‐30 according to the manufacturer's specifications. Ultra‐thin sections (70 nm) were cut with a diamond knife (Ultra 45°, DIATOME) and collected on copper grids (G300‐Cu, Electron Microscopy Sciences). Samples were observed by transmission electron microscopy (Talos L120C, Thermo Fisher Scientific) at 120 kV. Images were acquired with a digital camera (Ceta CMOS Camera, Thermo Fisher Scientific).

### 2.11 Molecular Dynamics Simulations of Wild‐Type and Mutant (Arg298Lys) *Hv*CHLI Models at 4°C and 22°C

Molecular dynamics (MD) simulations were performed using the models predicted in our previous work (Persello et al. 2024). Briefly, the homo‐hexameric ring model of the barley *Hv*CHLI ATPase subunit of Mg‐chelatase was determined using version 3 of AlphaFold2 Multimer from DeepMind (Evans et al. 2022). The predicted model was refined using the Protein Preparation Wizard from the Schrödinger Maestro suite (version 13.7.125, release 2023‐3; Madhavi Sastry et al. 2013). The Residue Scanning and Mutation module from the same suite was used to replace Arg298 with lysine (Arg298Lys; Beard et al. 2013). The FoldSeek web server was used to perform structural similarity analysis against the RCSB PDB100 database (Van Kempen et al. 2024; Berman et al. 2000). The hexameric structure of the chaperone Heat Shock Locus U (HSLU, PDB ID: 1DO0) from *E. coli* , solved by X‐ray crystallography, was selected on the basis of the presence of a magnesium ion and ATP in the binding cleft (Bochtler et al. 2000). The high degree of homology between the ATP‐binding domain of the chaperone HSLU and the *Hv*CHLI ATPase subunit allowed the distances between the magnesium ion, the ATP molecule, and the conserved residues in both proteins to be inferred. This information was used to introduce the magnesium ion into the predicted model, which enabled the ATP molecule to be docked into the binding cleft. The docking process was performed on both the wild‐type *Xan‐h* and the mutant *xan‐h.chli‐1* (Arg298Lys) models using GlideXP (Friesner et al. 2006). For each model, the most representative pose—in terms of the docking score—was deposited in ModelArchive, hosted by the Swiss Institute of Bioinformatics, with the accession codes ma‐xoqwu (*Xan‐h*) and ma‐tvik6 (*xan‐h.chli‐1*; Schwede et al. 2009). These two models were subsequently used to perform MD simulations. As our interest was focused on the interaction between residues Arg/Lys298 and the ATP molecule, only the *Hv*CHLI dimer defining the cleft (into which the magnesium ion was positioned and ATP was docked) was retained, while the other four monomers were removed from the models. To prevent the dimer from losing its original fold, positional restraints were applied to the backbone of residues at the monomer‐monomer interaction interface (excluding the interface to which ATP was docked) throughout all preparation and production steps. Specifically, for chain A (where the Arg/Lys298 residue protrudes toward the ATP in the model), the restrained residues encompass Gly151 to Val166, Asp225 to Phe255, and Leu313 to Phe412. For chain B, the residues involved in the positional restraints extend from Leu129 to Gly213, Val282 to Asp336, and Val379 to Phe412 (Figure S5A).

The wild‐type and the mutant *Hv*CHLI dimers were prepared using CHARMM‐GUI and the CHARMM36m force field (Jo et al. 2008; Brooks et al. 2009; Lee et al. 2016). Three independent MD simulations were conducted for each system at two temperatures: 4°C (277.15 K) and 22°C (295.15 K). The simulation workflow, performed with GROMACS version 2024.2, consisted of three stages: energy minimization, equilibration, and production (Van Der Spoel et al. 2005). The energy minimization step was performed using the steepest descent algorithm for a maximum of 5000 steps, with a convergence criterion of 500 kJ^−1^ /mol/^−1^ nm^−1^ for the force. Positional restraints were applied with force constants of 800 kJ/mol/nm^2^ for the backbone, 40 kJ/mol/nm^2^ for the side chains, and 1000 kJ/mol/nm^2^ for the backbone of the monomer‐monomer interface. A cut‐off of 1.2 nm was applied for both short‐range van der Waals and Coulomb interactions, while the Particle Mesh Ewald (PME) method was used for long‐range electrostatics (Petersen 1995). All bonds involving hydrogen atoms were constrained using the LINCS algorithm (Hess et al. 1997). The equilibration phase consisted of running a 100‐ns MD simulation with a 2‐fs time step. Positional restraints were applied with force constants of 400 kJ/mol/nm^2^ for the backbone, 40 kJ/mol/nm^2^ for the side chains, and 800 kJ/mol/nm^2^ for the backbone of the monomer‐monomer interface. Temperature coupling was maintained using the velocity‐rescale (v‐rescale) algorithm (Bussi et al. 2007) with separate coupling groups for the solute and solvent. The reference temperature was set to 277.15 or 295.15 K for both groups, with a velocity relaxation constant of 0.2 ps. Pressure coupling was applied using a semi‐isotropic stochastic cell rescaling (c‐rescale) barostat (Bernetti and Bussi 2020) with a time constant of 0.5 ps, a reference pressure of 1 bar, and a compressibility of 4.5 *×* 10^*−*5^ bar^*−*1^. Production MD simulations were performed for 2 μs with a 2‐fs time step, using the v‐rescale thermostat and the c‐rescale barostat with the same settings as used for the equilibration phase. Positional restraints with force constants of 700 kJ/mol/nm^2^ were applied exclusively to the backbone at the monomer‐monomer interface. The PME method was used to calculate long‐range electrostatic interactions. The LINCS algorithm was employed to constrain all bonds involving hydrogen atoms. Simulations were performed under periodic boundary conditions in the NPT ensemble.

In all, 24 μs of simulation trajectories were collected, each comprised of three replicates of 2 μs–that is, 6 μs at 277.15 K and 6 μs at 295.15 K for both the wild‐type *Hv*CHLI dimer and the mutant dimer. The simulations were visually inspected using Visual Molecular Dynamics (VMD) version 1.9.3 (Humphrey et al. 1996). The same tool was used to evaluate possible interactions between Arg/Lys298 and ATP, focusing on the ATP atoms N1, N3, N7, O2′, O3′, and O4′ (Figure S5B). The main interactions identified with VMD were further analyzed with PLUMED2 version 2.10 (Tribello et al. 2014). Specifically, for each frame of each wild‐type *Hv*CHLI dimer trajectory, the distances between the three nitrogen atoms (NE, NH1, NH2) connected to the carbon atom (CZ) at the tip of the arginine side‐chain and the nitrogen atom N3 of the adenine ring of ATP were calculated. The same calculations were performed between the nitrogen atoms of the arginine side‐chain and the oxygen atom of the ATP ribose ring (O4′). These distances were then used to assess the formation of hydrogen bonds between these moieties. An additional set of distances was calculated between the center of mass of carbon atoms C4 and C5 of the ATP adenine group (used as a proxy for the two aromatic rings) and the CZ atom of the arginine side‐chain. This latter distance was analyzed to evaluate the cation‐*π* interactions between ATP and the arginine side‐chain. For the mutant dimer, distances were calculated for each frame of the trajectory between the nitrogen atom (NZ) at the tip of the lysine side‐chain and the nitrogen N3 of the adenine ring of ATP. Similar calculations were performed between NZ and the O4′ atom of the ATP ribose ring. As for the wild type, an additional set of distances was calculated between the centers of mass of carbon atoms C4 and C5 of the ATP adenine group and the NZ atom of lysine; this allowed us to evaluate cation–*π* interactions between ATP and the lysine side‐chain. All atom‐atom distance plots were generated using custom Python 3 scripts based on the matplotlib, pandas, and numpy libraries (Van Rossum and Drake 2009; Hunter 2007; McKinney 2010; Harris et al. 2020). The atomic nomenclature used is based on the CHARMM force field (Figure S5). Relevant input files and trajectories are available on Zenodo (2013) under DOI: https://doi.org/10.5281/zenodo.14680350.

### 2.12 RNA Extraction, cDNA Synthesis and RT‐qPCR Expression Analysis

Total RNA was extracted from barley leaves as described in Verwoerd et al. 1989. For each sample, 1 μg of total RNA was retrotranscribed to cDNA using the iScript gDNA Clear cDNA Synthesis Kit (Bio‐Rad), following the manufacturer's procedures. The cDNA obtained was diluted 1:10. The quantitative Real‐Time PCR was set in a final volume of 12 μL adding 6 μL of SYBR master mix (Bio‐Rad), 5.2 μL of cDNA, and 0.5 μL of each primer (10 mM). The RT‐qPCR run was carried out using a CFX96 Real‐Time system (Bio‐Rad). The *HvActin7* gene was used as an internal control. Data from three biological and technical replicates were analyzed with the software Bio‐Rad CFX Maestro.

### 2.13 RNA‐Seq for Differential Gene Expression Analyses

For quantitative transcriptome analyses, total RNA was isolated from the middle leaf section of second‐leaf samples that had been grown under controlled greenhouse conditions for 14 days and were then shifted to 4°C for a further 25 days, together with control plants grown for 14 days under greenhouse conditions. Samples were obtained from five biological replicates, each composed by five plants of *Xan‐h* and *xan‐h.chli‐1* under both conditions. Differential gene expression analysis was performed in R using the DESeq2 package (Love et al. 2014). The biological replicates that fell outside the clusters in principal component analysis (PCA) were not considered during this analysis, but at least three replicates per condition were retained. Differentially expressed genes were filtered for a logFC > |0.5| and an adjusted *p*‐value < 0.05. Differential expression analysis was performed by comparing *xan‐h.chli‐1* to *Xan‐h*, both under cold‐stress induction (*xan‐h.chli‐1* 4°C vs. *Xan‐h* 4°C), *Xan‐h* under cold stress to *Xan‐h* under optimal greenhouse conditions (*Xan‐h* 4°C vs. *Xan‐h* 22°C) and *xan‐h.chli‐1* under cold stress to *xan‐h.chli‐1* optimal greenhouse conditions (*xan‐h.chli‐1* 4°C vs. *xan‐h.chli‐1* 22°C). For both *Xan‐h* 4°C versus *Xan‐h* 22°C and *xan‐h.chli‐1* 4°C versus *xan‐h.chli‐1* 22°C, only those genes found to be differentially expressed specifically in these comparisons were retained. An in‐house built script, based on the Monocot Plaza v4.5 (https://bioinformatics.psb.ugent.be/plaza/versions/plaza_v4_5_monocots/) database and the ncbi‐blast‐2.14.0+ tool (ftp.ncbi.nlm.nih.gov/blast/executables/blast+/LATEST/), was used to identify the orthologous Arabidopsis genes. Subcellular localization was predicted with SUBA5 (suba.live/index.html). Biological Process Gene Ontology (GO) term enrichment was performed with the R package gprofiler2 (Kolberg et al. 2020) by filtering for more than 10 genes associated to the GO term and an FDR < 0.01. Subcellular protein localization and chloroplast transit peptide (cTP) predictions were identified by TargetP (shttps://services.healthtech.dtu.dk/ services/TargetP‐2.0/).

## 3 Results

### 3.1 The *xan‐h.chli‐1* Mutant Exhibits a Distinct Cold‐Sensitive Phenotype

**FIGURE 1:**
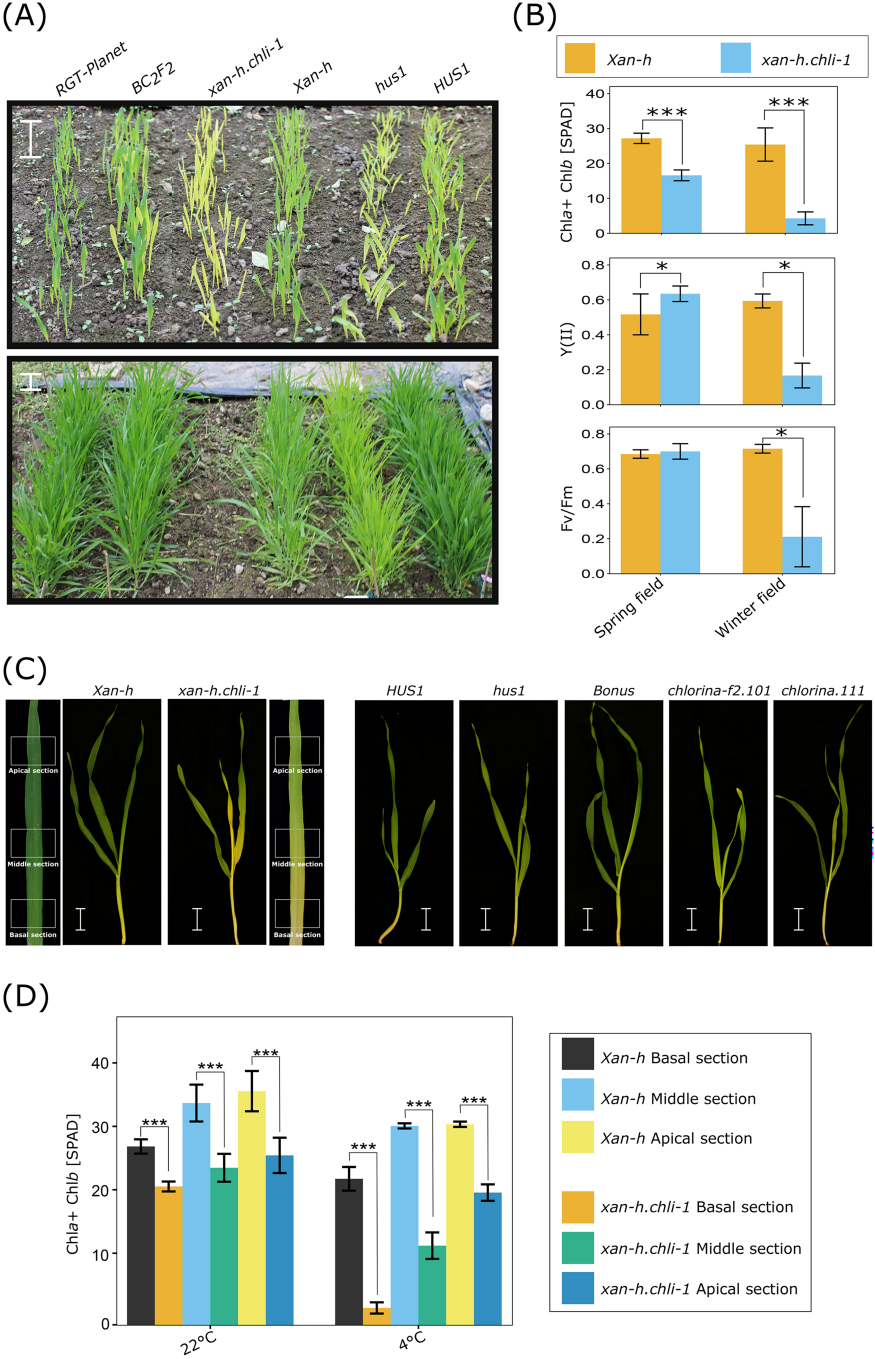
Cold‐sensitive phenotypes of the *xan‐h.chli‐1* mutant grown either in the field or under controlled growth‐chamber conditions (14 days at 22°C, 25 days at 4°C). (A) *xan‐h.chli‐1* and *hus1* mutant plants, together with *Xan‐h* and *HUS1* wild‐type controls, were grown in the field during the winter season 2023–2024 in rows of 80 seeds each genotype. The *BC*_2_ *F*_2_ generation of *xan‐h.chli‐1* was also backcrossed with the elite cultivar *RGT‐Planet* and included in the field trial, together with the wild‐type *RGT‐Planet* as a control. Cold‐sensitive lethal phenotypes of barley seedlings were photographed at the two‐leaf stage (upper panel) and the tillering stage (lower panel), 32 and 102 days after sowing (DAS), respectively. The images shown were taken on 24/01/2024 (see also Figure S1) and 08/03/2024. The *xan‐h.chli‐1* plants did not survive the winter season, as shown by the absence of plants in the third row in the bottom panel. The same cold‐induced *xan‐h.chli‐1* lethal phenotype was observed in the winter season 2022–2023. Scale bar: 5 cm. (B) Measurements of total leaf chlorophyll content and the photosynthetic parameters Y(II) and Fv/Fm of *Xan‐h* and *xan‐h.chli‐1* plants at the second‐leaf stage. The data were obtained with the SPAD‐502 chlorophyll meter and the Handy PEA fluorometer, respectively, in barley plants grown in winter (15/01/2023; 07/01/2024) and spring (12/04/2023; 14/04/2024) field conditions. Nine plants per genotype/condition were analysed. (C) Visible phenotypes of leaves from *xan‐h.chli‐1*, *hus1* (*Hvcpsrp43*), *chlorina‐f2.101* (*Hvcao*) and *chlorina.111* (*Hvcrd1*) mutant plants were compared with those obtained for *Xan‐h*, *HUS1* and *Bonus* control plants that had been adapted to cold‐stress conditions. Basal, Middle and Apical sections are shown. Scale bar: 2 cm. (D) Comparisons of total chlorophyll contents of second leaves of *xan‐h.chli‐1* and *Xan‐h* control plants that had been exposed to cold stress or grown under optimal greenhouse conditions. Six/eight plants per genotype/condition were analysed. Student's *t*‐test was performed to determine the significance of the observed differences (***p < 0.001, **p < 0.01, *p < 0.05).

The pale‐green barley line *xan‐h.chli‐1* was previously identified as the only viable mutant available at the essential *Xan‐h* locus, which codes for subunit I of Mg‐chelatase (Persello et al. 2024). To investigate its physiological performance and evaluate plant productivity under field conditions, wild‐type *Xan‐h* control plants (cv. Morex) and *xan‐h.chli‐1* mutant plants were sown in November 2022 and 2023 at the Botanical Garden “Città Studi” in Milan (Italy). Two independent field trials were conducted, which also included the pale‐green mutant *hus1*, defective in the chloroplast signal recognition particle 43 kDa (*Hv*cpSRP43) and its corresponding control, *HUS1* (Rotasperti et al. 2022). In the second winter trial, the segregating F2 population (*BC*_*2*_ *F*_*2*_), obtained by backcrossing *xan‐h.chli‐1* with cv. *RGT‐Planet*, was also included, and the wild‐type *RGT‐Planet* served as the control. Notably, *xan‐h.chli‐1* plants and their homozygous mutant siblings (*xan‐h.chli‐1/xan‐h.chli‐1*) within the *BC*_*2*_ *F*_*2*_ population displayed pronounced sensitivity to winter conditions immediately after germination, which ultimately led to the death of the seedlings (Figure 1A and Figure S1). This winter‐induced lethality was associated with a dramatic reduction in leaf chlorophyll content, as evidenced by a yellow‐leaf phenotype and significantly reduced photosynthetic efficiency of *xan‐h.chli‐1* (Figure 1B). Photosynthetic performance was measured as the maximum quantum yield of PSII (Fv/Fm) and the effective quantum yield of PSII (Y[II]; Figure 1B). In contrast to these findings, wild‐type‐like siblings within the *BC*_*2*_ *F*_*2*_ segregating population, along with all other genotypes grown under the same field conditions, were able to withstand winter and complete their life cycles. Interestingly, the lethality of *xan‐h.chli‐1* plants was strictly linked to winter conditions. No such mortality was observed during spring field trials, conducted with the same set of plants sown in March 2022 and 2023, or under optimal greenhouse conditions. These findings suggest that the winter‐induced lethal phenotype may result from an exacerbated sensitivity to cold temperatures (Figure 1B, Figure S1).

To further investigate this phenotypic feature, *Xan‐h* and *xan‐h.chli‐1* plants were grown under greenhouse conditions for 14 days and subsequently exposed to 4°C for 25 days to induce cold stress. To determine whether cold sensitivity is a common characteristic of the pale‐green trait in barley, additional mutant lines with defects in various steps of chlorophyll biosynthesis or antenna protein biogenesis were included as controls. These lines included *chlorina‐f2.101* and *chlorina.111*, impaired in chlorophyllide‐a oxygenase (*Hvcao*) and the catalytic component of the MgProto monomethyl ester (*MgProtoME*) cyclase complex (*Hvcrd1*), respectively (Bossmann et al. 1997; Simpson et al. 1985), as well as *hus1* (Rotasperti et al. 2022; Table S1).

As expected, exposure to cold stress led to a general reduction in growth rate across all plant lines. However, only in the case of *xan‐h.chli‐1*, a distinct gradient of chlorosis was observed in younger leaf tissues upon cold exposure (Figure 1C). Both the cold‐exposed pale‐green mutants (*hus1*, *chlorina‐f2.101*, and *chlorina.111*) and the wild‐type control lines *Xan‐h*, *HUS1*, and *Bonus* retained relatively uniform leaf pigmentation. Specifically, in *xan‐h.chli‐1*, three distinct phenotypic sections could be identified in the second leaf based on chlorophyll content compared to wild‐type *Xan‐h* controls (Figure 1C,D): (i) a severely chlorotic basal section in the proximal area of the emerging leaf, (ii) an intermediate chlorotic phenotype in the middle section, and (iii) a milder pale‐green coloration in the apical section, similar to that of *xan‐h.chli‐1* leaves grown under optimal greenhouse conditions (Persello et al. 2024). Interestingly, this leaf chlorosis phenotype was absent both in adult *xan‐h.chli‐1* plants exposed to high light intensities under spring/summer field conditions (Figure S1) and in seedlings grown under controlled high‐light and optimal temperature conditions (Figure S2). Under such conditions, *xan‐h.chli‐1* second leaves exhibited higher photosynthetic efficiency compared to *Xan‐h*, as previously reported (Persello et al. 2024). Collectively, these observations suggest that the cold‐sensitive phenotype specifically observed in the *xan‐h.chli‐1* mutant is associated with the amino‐acid substitution Arg298Lys in the *Hv*CHLI subunit of Mg‐chelatase and is not a general phenomenon associated with reduced chlorophyll content as such.

This finding was further validated by growing *Arabidopsis thaliana* *Atchli1* knockout mutants transformed with the barley *Xan‐h* and *xan‐h.chli‐1* allelic variants (*Atchli1/Atchli1+35S::Xan‐h* and *Atchli1/Atchli1+35S::xan‐h.chli‐1*, respectively; Persello et al. 2024) at 4°C under controlled conditions. Both Arabidopsis Col‐0 and *Atchli1/Atchli1+35S::Xan‐h* plants exhibited a wild‐type, cold‐tolerant phenotype, while the photosynthetic performance of *Atchli1/Atchli1+35S::xan‐h.chli‐1* leaves was significantly reduced after prolonged cold exposure (Figure S3A), mimicking the response observed in the *xan‐h.chli‐1* barley mutant. Interestingly, the Arabidopsis mutant line *cs/cs*, which carries a modified CHLI subunit that leads to an extended C‐terminal region (Kobayashi et al. 2008; Koncz et al. 1990) exhibited little to no sensitivity to cold treatment (Figure S3A,B), despite the fact that the CHLI protein in *cs/cs* leaves accumulates to a significantly lower extent than that found in *Atchli1/Atchli1+35S::xan‐h.chli‐1* plants (Figure S3C).

### 3.2 Recovery of the *xan‐h.chli‐1* Cold‐Sensitive Phenotype in Young Developing Tissues Under Optimal Temperature Conditions

**FIGURE 2:**
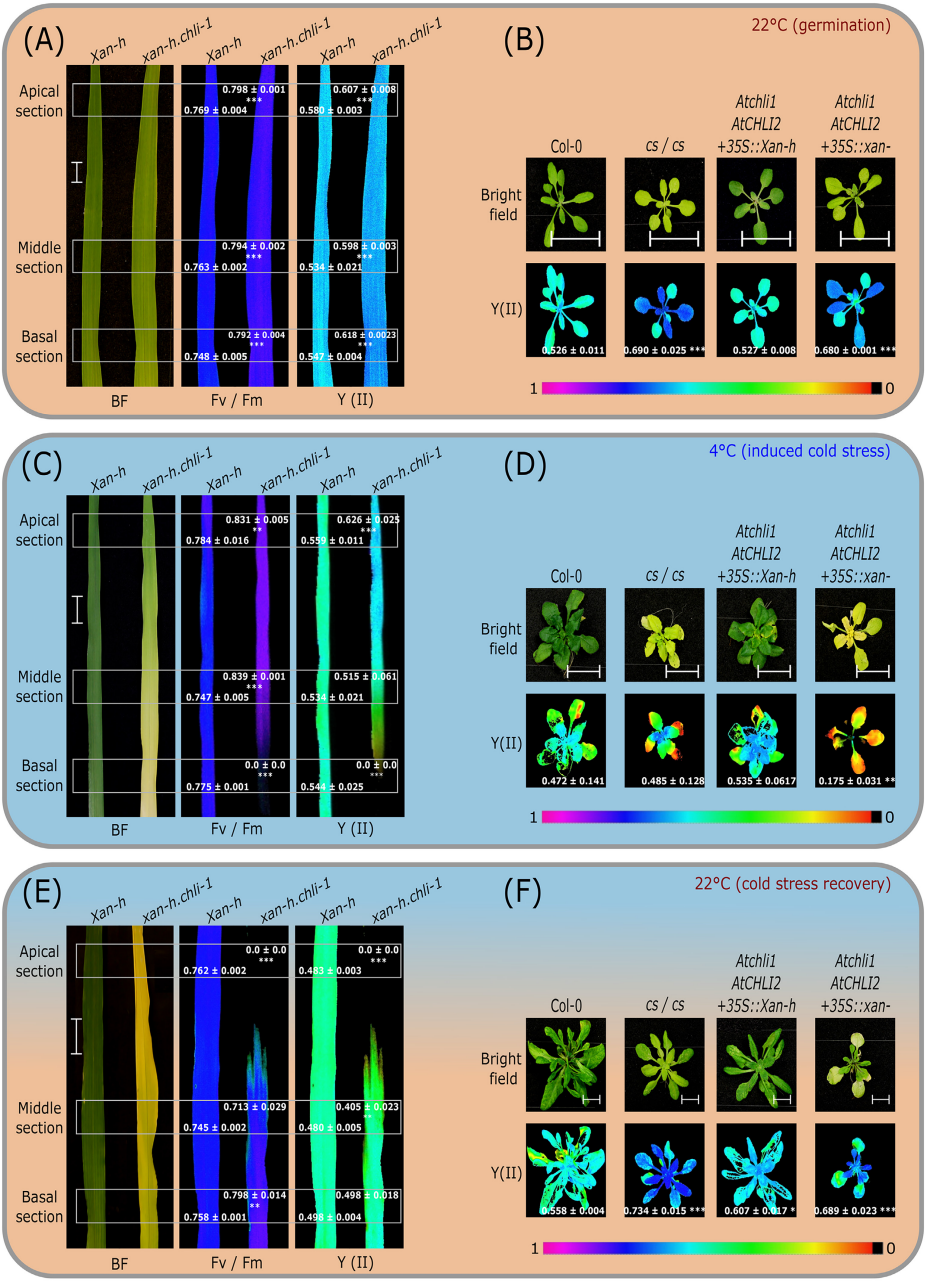
Effects of cold stress without (14 days at 22°C, 25 days at 4°C) and with a recovery period (14 days at 22°C, 25 days at 4°C, and 5 days at 22°C) on the photosynthetic performance of barley and *A. thaliana* plants homozygous for *xan‐h.chli‐1*, together with control plants. (A) Visible phenotype (bright field illumination, BF) of barley *xan‐h.chli‐1* leaves compared to those of *Xan‐h* after 14 days under greenhouse control conditions, together with PSII photosynthetic parameters Fv/Fm and Y(II). (B) Visible phenotypes and PSII photosynthetic performance of *Atchli1/Atchli1* plants that had been transformed with *Xan‐h* and *xan‐h.chli‐1* coding sequences, and the corresponding controls Col‐0 and *cs/cs* genotypes under control conditions. (C) Visible phenotypes of *Xan‐h* and *xan‐h.chli‐1* leaves grown under induced cold‐stress conditions, together with PSII photosynthetic parameters Fv/Fm and Y(II). (D) Arabidopsis plants as in (B) following exposure to cold stress, together with PSII photosynthetic parameter Y(II). (E) Visible phenotypes and PSII photosynthetic parameters of *Xan‐h* and *xan‐h.chli‐1* upon recovery from cold conditions, together with PSII photosynthetic parameters Fv/Fm and Y(II). (F) Arabidopsis plants as in (B) and (D) following recovery from exposure to cold, together with PSII photosynthetic parameter Y(II). The photosynthetic parameters Fv/Fm and Y(II) are presented in false‐colour images, representing average values obtained from three independent leaves per rosette. A total of 10 plants per genotype were analysed. The colour scale is shown below the images, with violet corresponding to 1 and red to 0. Scale bar: 2 cm. Student's *t*‐test was performed to show the significance of the observed differences (***p < 0.001, **p < 0.01, *p < 0.05).

The gradual decrease in chlorophyll levels observed under induced cold‐stress over the entire length of growing *xan‐h.chli‐1* leaves correlates with a significant decline in photosynthetic performance, as indicated by reduced Fv/Fm and Y(II) values relative to control conditions (Figure 2A,C). Notably, in the basal leaf section of cold‐stressed mutants, chlorophyll fluorescence was undetectable, and PSII activity (Fv/Fm and Y[II]) was absent. Conversely, the middle and apical sections displayed higher PSII performance than controls, in accordance with observations made under optimal growth conditions (Persello et al. 2024). To assess recovery, cold‐adapted plants were returned to optimal greenhouse conditions, and photosynthetic efficiency was re‐evaluated after 5 days (Figure 2E). During this cold‐stress recovery phase, *xan‐h.chli‐1* plants regenerated photosynthetically functional tissues. Specifically, the basal and middle leaf sections exhibited Fv/Fm and Y(II) values that exceeded those seen in the *Xan‐h* control and resembled plants grown under optimal conditions. However, the yellow apical section of *xan‐h.chli‐1* leaves showed visible deterioration, with photosynthetic efficiency parameters no longer detectable.

Similar patterns were observed in transformed Arabidopsis *Atchli1/Atchli1+35S::xan‐h.chli‐1* lines subjected to cold treatment in respect to *Col‐0* and the *cs/cs* mutant, in which the *AtCHLI1* fusion protein is seven amino acids longer than its wild‐type counterpart (Koncz et al. 1990) and accumulates to much lower levels than does the barley *xan‐h.chli‐1* variant (Figure S3). While *Col‐0*, *cs/cs*, and *Atchli1/Atchli1+35S::Xan‐h* lines exhibited a comparable reduction in photosynthetic parameters, Y(II) values were severely reduced in cold‐exposed *Atchli1/Atchli1+35S::xan‐h.chli‐1* plants (Figure 2B,D). However, upon renewed exposure to optimal growth temperatures, all plant lines exhibited a general recovery of photosynthetic functionality (Figure 2F). This recovery suggests that the defects induced by transient low‐temperature exposure in plants carrying the *xan‐h.chli‐1‐*coding sequence can be mitigated, at least in young, developing leaf tissues (Figure 2E,F).

### 3.3 Cold Stress Induces Alterations in Chloroplast Ultrastructure in *xan‐h.chli‐1* Leaves

**FIGURE 3:**
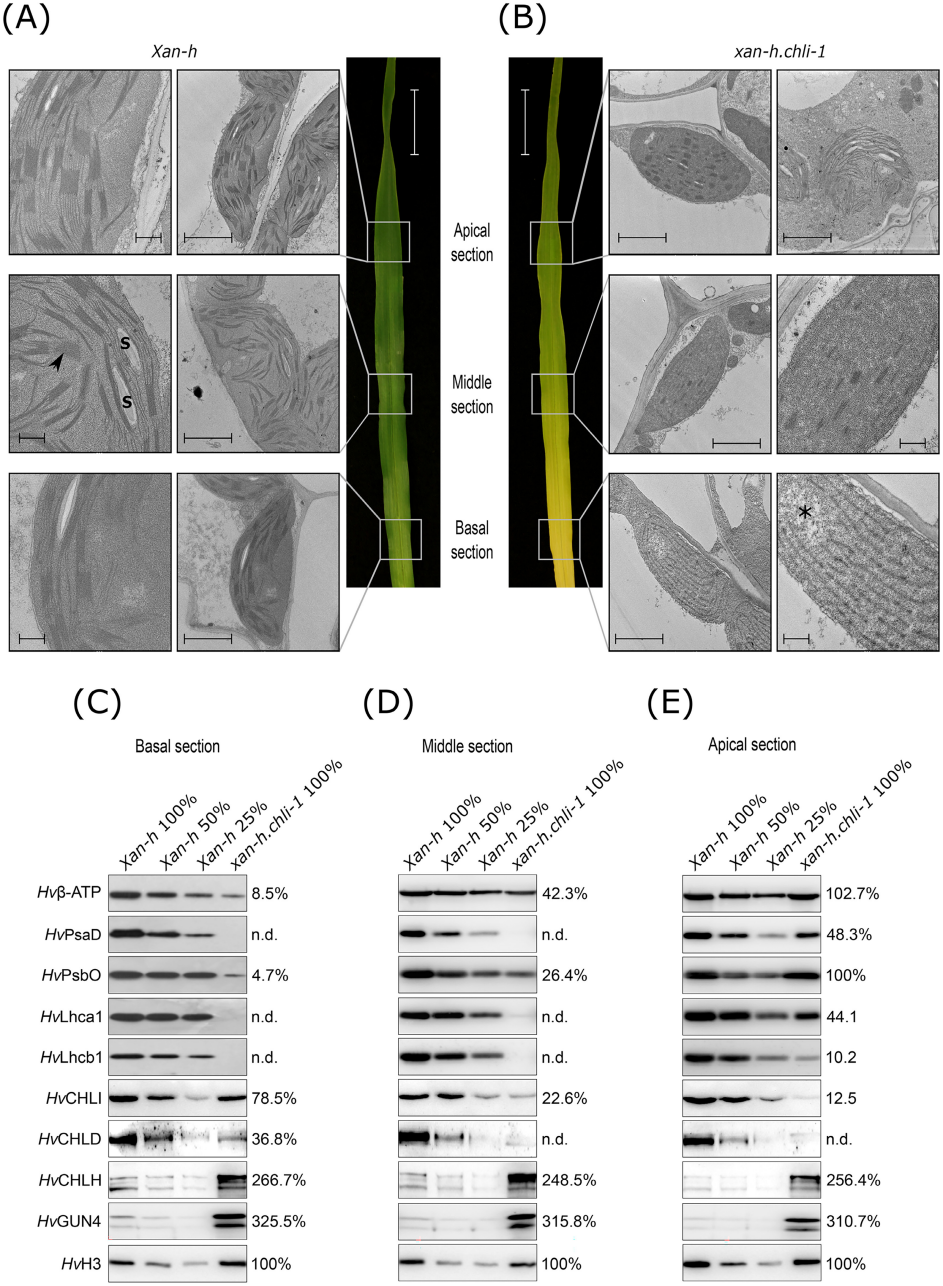
Structural and biochemical characterization of plastid development in *xan‐h.chli‐1* and *Xan‐h* plants that had been exposed to cold‐stress conditions (14 days at 22°C, 25 days at 4°C). (A‐B) Representative TEM micrographs (30 micrographs for each section have been collected) showing chloroplast ultrastructure in basal, middle and apical sections of second leaves of *Xan‐h* (A) and *xan‐h.chli‐1* (B) plants. S, starch granule; arrowheads, thylakoid grana stacks; asterisk, plastid nucleoids; Scale bar = 1 um for TEM images and scale bar = 2 cm for leaf immages. (C–E) Immunoblot analyses of plastid‐located proteins from *Xan‐h* and *xan‐h.chli‐1* basal (C), middle (D) and apical leaf (E) sections developed under cold stress conditions. Protein extracts were probed with antibodies specific for thylakoid electron transport chain subunits (*Hv*β‐ATP, *Hv*PsaD, *Hv*PsbO, *Hv*Lhca1 and *Hv*Lhcb1) and Mg‐chelatase subunits (*Hv*CHLI, *Hv*CHLD, *Hv*CHLH, *Hv*Gun4). Immunoblots on the barley histone H3 protein were used as loading control. One of three biological replicates is shown. The percentage values next to each immunoblot indicate the relative protein abundance in *xan‐h.chli‐1* leaf samples, expressed as a percentage of the control (*Xan‐h*), which is set to 100%.

Plastid ultrastructure was analyzed using transmission electron microscopy in the leaves of *Xan‐h* and *xan‐h.chli‐1* plants exposed to cold stress conditions (Figure 3). The leaf blade of *Xan‐h* primarily displayed fully developed chloroplasts with well‐formed thylakoids, similar to those observed under optimal growth conditions (Figure 3A; Persello et al. 2024). In contrast to this finding, the *xan‐h.chli‐1* leaf blade exhibited plastids with varying shapes and alterations in thylakoid membrane organization (Figure 3B). Specifically, the basal section of *xan‐h.chli‐1* leaves contained proplastid‐like organelles characterized by thylakoids lacking grana stacks and regions of low electron density resembling plastid nucleoids (Krupinska et al. 2014). In the middle section, young, developing chloroplasts with few grana stacks and minimal starch accumulation were observed. The apical section of *xan‐h.chli‐1* leaves under cold stress contained two types of plastids: (i) mature chloroplasts with reduced grana stacks and limited starch accumulation, together with (ii) burst chloroplasts marked by swollen thylakoid membranes and loss of plastid envelope integrity (Figure 3B).

The organization of plastid thylakoid membranes closely correlated with the assembly of the photosynthetic machinery within thylakoids. In the basal and middle sections of *xan‐h.chli‐1* leaves, the levels of subunits of the thylakoid protein complexes were significantly reduced relative to the control (Figure 3C,D). These include antenna proteins of photosystem I (PSI; *Hv*Lhca1) and PSII (*Hv*Lhcb1), the beta subunit of ATP synthase (*Hv*β‐ATP), the *Hv*PsbO subunit of the oxygen‐evolving complex, and the *Hv*PsaD subunit of the PSI core. In the apical section, however, the levels of these subunits were higher and could reach concentrations between those of the control and mutant leaves (Figure 3E; Persello et al. 2024). Specifically, with regard to Mg‐chelatase subunits, the accumulation of *Hv*CHLH and *Hv*Gun4 was higher in mutant chloroplasts than in the control across all three sections of the cold‐adapted leaf. Conversely, relative to *Xan‐h*, *Hv*CHLI and *Hv*CHLD levels progressively decreased from the basal to the apical sections of *xan‐h.chli‐1* leaves (Figure 3C–E).

To further explore the developmental and physiological characteristics of plastids in different sections of the *xan‐h.chli‐1* leaf blade, RT‐qPCR was performed on marker genes associated with proplastid‐containing tissues, such as *HvSCA3* and *HvWRKY40* (Van Aken et al. 2013; Liu et al. 2013; Hricová et al. 2006; Emanuel et al. 2004), as well as stress‐related genes (*HvHSFA4A* and *HvLOX2*; Pérez‐Salamó et al. 2014; Bell et al. 1995), including two ROS‐scavenger‐encoding genes (*HvFLOT3* and *HvCAT1*; Junková et al. 2018; Méndez‐Gómez et al. 2024; Xing et al. 2008), across the three leaf sections (Figure S4A). *HvSCA3* and *HvWRKY40* exhibited similar expression patterns in *xan‐h.chli‐1*, with higher overall expression compared to *Xan‐h* across all leaf sections. *HvHSFA4A* and *HvLOX2* were more highly expressed in the middle and apical sections, while *HvFLOT3* showed a similar pattern to *HvSCA3* and *HvWRKY40*. Notably, relative to *Xan‐h*, *HvCAT1* expression was predominantly observed in the apical section of *xan‐h.chli‐1*. These findings indicate that in *xan‐h.chli‐1*, the basal section of the leaf primarily contains proplastids or juvenile chloroplasts.

**FIGURE 4:**
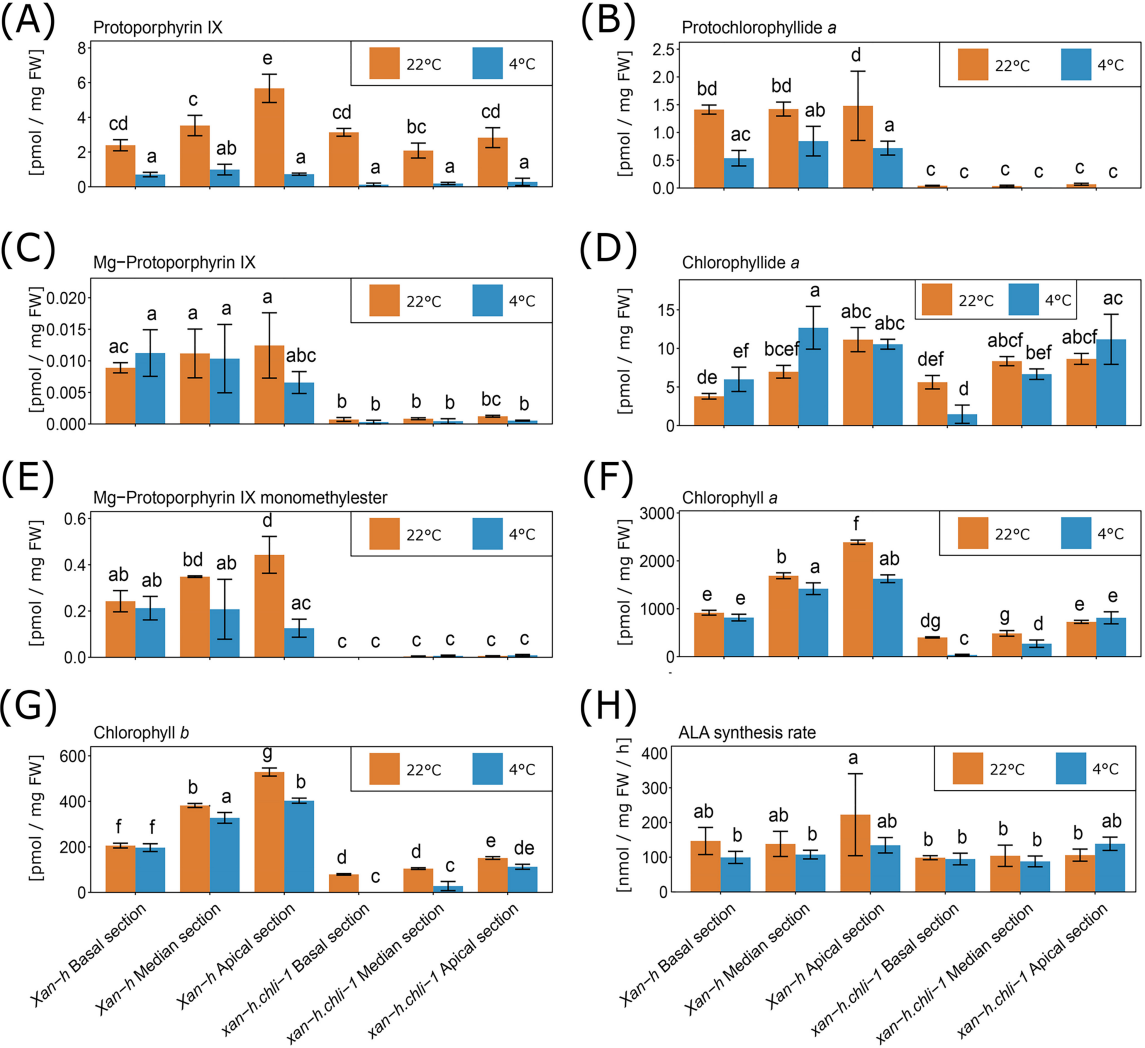
Chlorophyll intermediate and ALA synthesis rate analysis under control (22°C) and cold (4°C) conditions. (A–G) Steady state levels of chlorophyll intermediates such as protoporphyrin IX (A), protochlorophyllide *a* (B), Mg‐protoporphyrin IX (C), chlorophyllide *a* (D), Mg‐protoporphyrin IX monomethylester (E), chlorophyll *a* (F) and chlorophyll *b* (G) in the three sections of the second leaf of *xan‐h.chli‐1* and *Xan‐h*. The leaf samples were harvested from seedlings, which grew 14 days at 22°C (control condition) and then 25 days at 4°C (cold stress condition). (H) Analysis of the 5‐aminolevulinic acid (ALA) synthesis rate from *Xan‐h* and *xan‐h.chli‐1* seedlings grown for 1 week at 22°C (control condition) or for 1 week at 22°C and then transferred to 4°C for another week (cold stress condition). Values are the average of five biological replicates. Letters indicate Anova classification based on Tukey's HSD test (Tukey's *p*‐value < 0.05).

Along the chlorophyll gradient, chloroplasts differentiate under suboptimal conditions, particularly in the apical section. Nevertheless, ROS accumulation measured over the whole leaves was lower in *xan‐h.chli‐1* samples than in *Xan‐h* after 10 days at 4°C (Figure S4B,C). Comparison of chlorophyll and intermediates of the tetrapyrrole biosynthetic pathway between wild‐type *Xan‐h* and *xan‐h.chli‐1* plants grown either in control conditions (14 days at 22°C) or induced cold conditions (14 days at 22°C and then 25 days at 4°C) revealed generally reduced contents of leaf pigments and their metabolic precursors in both lines after cold treatment (Figure 4A–G). Moreover, *xan‐h.chli‐1* leaves showed a marked reduction in the accumulation of chlorophyll biosynthetic intermediates in comparison to *Xan‐h*. Protoporphyrin IX steady‐state levels are reduced compared to wild type, while Mg‐protoporphyrin IX, Mg‐protoporphyrin IX monomethyl ester, and protochlorophyllide a levels of the mutant are close to the detection limit in all three leaf sections compared to *Xan‐h* (Figure 4A–G) Interestingly, the steady‐state concentrations of chlorophyllide *a* gradually increased from the basal to the apical region of *xan‐h.chli‐1* leaves, with a steeper increase in accumulating chlorophyllide after cold treatment. It can be speculated that chlorophyllide in older cells may not only be an intermediate of chlorophyll synthesis but also of the chlorophyll recycling pathway, which is emphasized by the light green phenotype (Lin et al. 2022). However, it currently remains unclear why chlorophyllide accumulation appears to be increased during cold treatment (Lin et al. 2022). The extremely low steady‐state concentrations of Mg‐porphyrins and protochlorophyllide *a* downstream of the Mg‐chelatase are explained by an impaired enzymatic chelate reaction. Since lower levels of protoporphyrin IX were also detected in *xan‐h.chli‐1* despite the mutation in *Hv*CHLI and an expected reduced Mg‐chelatase activity, it is hypothesized that the rate‐limiting step of 5‐aminolevulinic acid (ALA) is feedback‐controlled. The in vivo analysis of ALA synthesis rates across the three leaf sections reveals that the oldest section (the apical part) of wild‐type leaves exhibits the highest ALA synthesis rate (Figure 4H). Following cold treatment, ALA synthesis in the wild type is reduced overall but still shows a gradual increase from the base to the tip of the leaf. In contrast, the baseline ALA synthesis rate in the mutant is significantly lower than in the wild type, suggesting a previously uncharacterized feedback effect of the impaired Mg‐chelatase on the enzymes involved in ALA biosynthesis. Notably, this already reduced ALA synthesis capacity in the mutant does not decline further under cold conditions (Figure 4H).

The in vivo analysis of the three leaf sections of the ALA synthesis rate reveals that in the relatively young leaves, nevertheless, the older third part of the wild type leaves (the apical section) has the highest ALA synthesis rate. The ALA synthesis is reduced after cold treatment in the wild type, but still gradually increases toward the leaf tip. The control synthesis rate of the mutant is significantly reduced compared to the wild type. This suggests an as yet unknown feedback effect of the impaired Mg‐chelatase due to the *xan‐h.chli‐1* mutation on the enzymes of ALA synthesis. However, the lower ALA synthesis capacity of the mutant is no longer reduced in the cold (Figure 4H).

### 3.4 Molecular Dynamics Simulations Reveal Temperature‐Dependent Interactions Between Lys298 and ATP

To further investigate the cold‐sensitive phenotype of *xan‐h.chli‐1* plants, molecular dynamics (MD) simulations were performed with the predicted models of wild‐type *Hv*CHLI and its mutant variant (Arg298Lys) as starting conformations. Simulations were conducted under two temperature conditions, 4°C (277.15 K) and 22°C (295.15 K). To explore the molecular mechanisms underlying the Arg‐to‐Lys substitution, MD simulations focused on a single *Hv*CHLI dimer for both the wild‐type and mutant variants. The dimer interface forms the ATP‐binding cavity, with the Arg/Lys298 side‐chain extending toward the nucleotide. To maintain the dimer in its hexameric ring conformation, positional restraints were applied as described in the Materials and Methods section (Figure S5A). Each system was simulated in triplicate for 2 μs at both temperatures, resulting in a total simulation time of 24 μs.

The trajectories were analyzed using Visual Molecular Dynamics (VMD; Humphrey et al. 1996) to identify key interactions between ATP and the Arg/Lys298 side‐chain. Hydrogen bonds were observed between these residues and either the N3 atom of the adenine ring or the O4′ atom of the ribose ring. In addition, simulations revealed the possibility of cation‐π interactions between the aromatic adenine ring of ATP and the positively charged Arg/Lys298 side chain. A comprehensive analysis of all simulation frames was conducted using PLUMED (Tribello et al. 2014), focusing on the distances between the Arg/Lys298 side‐chain and atoms in ATP that had been identified during visual inspection.

**FIGURE 5:**
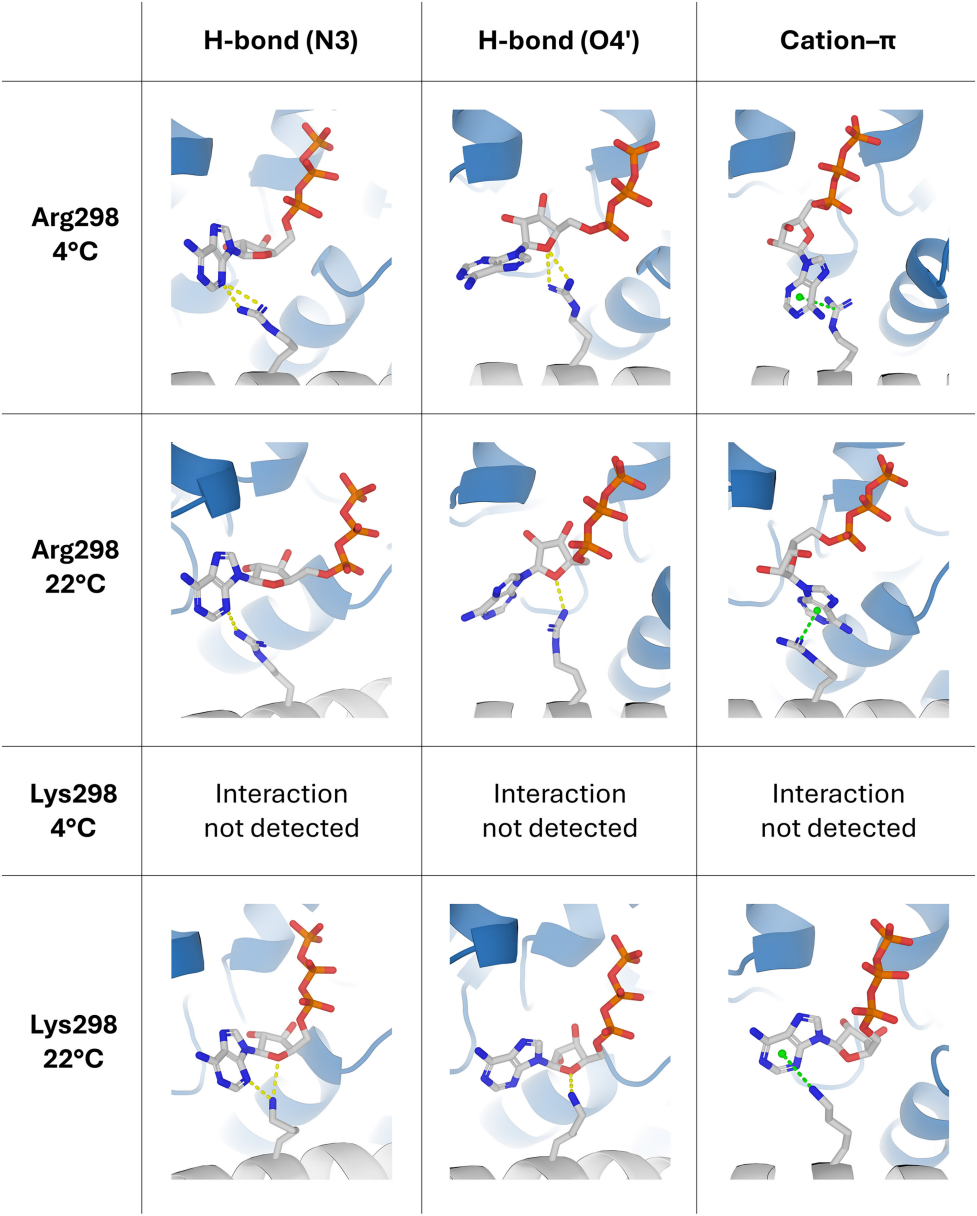
Main interactions between Arg/Lys298 and ATP at 4°C and 22°C in molecular dynamics simulations. The frames are representative of the interactions observed during MD simulations between Arg298 or Lys298 and ATP at two different temperatures: 4°C (277.15 K) and 22°C (295.15 K). No interactions between Lys298 and ATP were observed at 4°C. Arg298 and Lys298 (chain A) are shown as sticks with carbon (C) atoms in light grey, oxygen (O) atoms in red, and nitrogen (N) atoms in blue. The ATP molecule, with a net charge state of −4, is represented by sticks with C atoms in light grey, O atoms in red, N atoms in blue and phosphorus (P) atoms in orange. The hydrogen bonds between the side‐chains of Arg/Lys298 and ATP (atoms N3 or O4’) are shown as yellow dashed lines. The cation‐*π* interactions between the side‐chains of Arg/Lys298 and the adenine ring of ATP are shown as green dashed lines. The hydrogen atoms of Arg/Lys298 and ATP and the magnesium ion interacting with ATP are not shown.

Regardless of the temperature, the NE, NH1, and NH2 atoms of the Arg298 side‐chain remained sufficiently close to the N3 atom (Figure 5, Figures S6A, S7A) or the O4′ atom (Figure 5, Figures S6B, S7B) of ATP to establish hydrogen bonds. Arg298 was also capable of engaging in cation‐π interactions with the adenine ring at both temperatures (Figure 5, Figure S10A). In contrast to these findings, while Lys298 formed similar hydrogen bonds and cation‐π interactions at 22°C, these interactions were less frequent and less stable relative to those established by Arg298 (see also Persello et al. 2024). Specifically, at 22°C, the NZ atom of Lys298 could establish hydrogen bonds with the N3 atom (Figure 5, Figure S9A) or the O4′ atom (Figure 5, Figure S9B) of ATP and engage in cation‐π interactions with the adenine ring (Figure 5, Figure S10B). Notably, at 4°C, no interactions between Lys298 and ATP were observed (Figure 5, Figures S8, S10B).

### 3.5 Transcriptomic Analysis Highlights the Increased Sensitivity of *xan‐h.chli‐1* Leaves to Low Temperatures

The middle leaf section of *xan‐h.chli‐1* represents an intermediate physiological and chloroplast differentiation stage between the basal and apical sections. This section was characterized by moderate chlorophyll accumulation, thylakoid protein levels, photosynthetic performance, and plastid differentiation (Figures 1, 2, 3, 4). To investigate the long‐term effects of cold exposure on nuclear gene expression and to dissect the molecular mechanisms underlying plant tolerance and sensitivity, transcriptomic analyses were performed on the middle section of the second leaves from *Xan‐h* and *xan‐h.chli‐1* plants exposed to cold stress (4°C for 25 days under long‐day conditions). The results were compared to transcriptomic data from *Xan‐h* and *xan‐h.chli‐1* plants grown under optimal greenhouse conditions (22°C; Persello et al. 2024).

**FIGURE 6:**
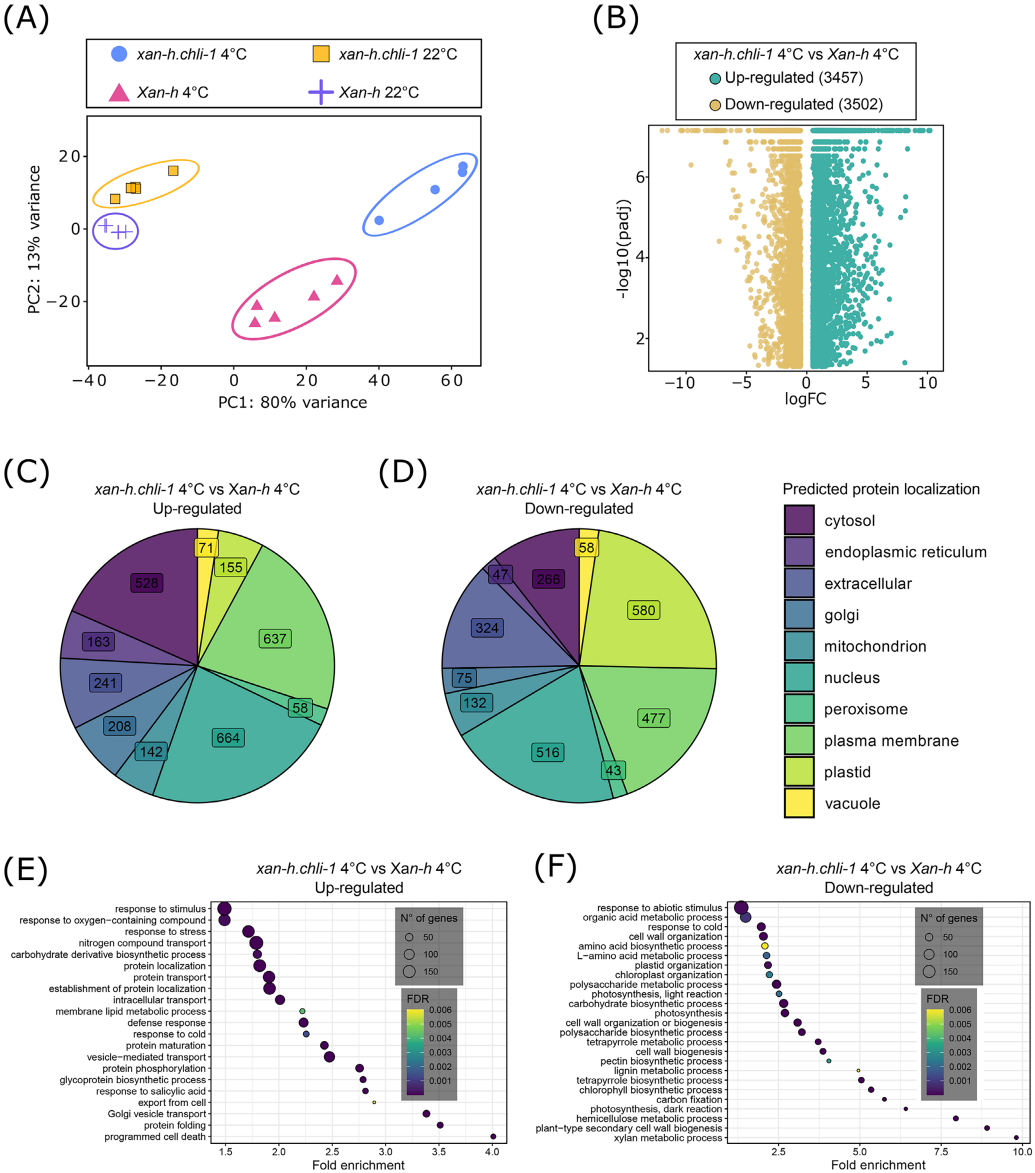
Transcriptomic and gene ontology (GO) enrichment analysis of *xan‐h.chli‐1* and *Xan‐h* second leaves under induced cold stress (14 days at 22°C, 25 days at 4°C) and control conditions (14 days at 22°C). Five biological replicates for each genotype/condition were analysed, a *xan‐h.chli‐1* replicate at 4°C was excluded from the analysis. (A) Principal component analysis (PCA) of the RNA‐Seq data. (B) Volcano plot showing the differentially expressed genes (DEGs) in the middle leaf section of *xan‐h.chli‐1* mutant plants under cold conditions compared to *Xan‐h* under cold conditions (*xan‐h.chli‐1* 4°C vs. *Xan‐h* 4°C) after filtering (padj < 0.05 and logFC *> |*0.5*|*). (C, D) Predicted subcellular localization of the barley *xan‐h.chli‐1* 4°C vs. *Xan‐h* 4°C DEGs homologs in *A. thaliana* for the up‐ (C) and down‐regulated genes (D). (E, F) A set of representative enriched (*p <* 0.01, number of genes > 10) biological process GO terms for the up and down regulated DEGs in the *xan‐h.chli‐1* 4°C versus *Xan‐h* 4°C comparison. The complete list is provided in Table S2.

Principal component analysis (PCA) revealed distinct clustering for each genotype and condition, forming four separate groups (Figure 6A). Notably, *Xan‐h* and *xan‐h chli‐1* clusters at 4°C diverged more with respect to the control clusters, indicating that the response to the cold treatment was markedly different in the two genotypes (Figure 6A). Three key comparisons were conducted: (1) *xan‐h.chli‐1* under cold stress vs. *Xan‐h* under cold stress (*xan‐h.chli‐1* 4°C vs. *Xan‐h* 4°C), (2) *Xan‐h* under cold stress vs. *Xan‐h* under optimal conditions (*Xan‐h* 4°C vs. *Xan‐h* 22°C), and (3) *xan‐h.chli‐1* under cold stress vs. *xan‐h.chli‐1* under optimal conditions (*xan‐h.chli‐1* 4°C vs. *xan‐h.chli‐1* 22°C). Differentially expressed genes (DEGs) were identified based on log‐fold change (logFC > |0.5|) and adjusted *p*‐value (padj < 0.05; Figure 6B). Protein localization prediction of *A. thaliana* homologs of the DEGs, along with Biological Process Gene Ontology (GO) enrichment analysis, was also performed (Table S2).

The *xan‐h.chli‐1* 4°C vs. *Xan‐h* 4°C comparison identified 3457 up‐regulated and 3502 down‐regulated genes in *xan‐h.chli‐1* relative to *Xan‐h* (Figure 6B). Most upregulated genes were predicted to be active in the nucleus (664), plasma membrane (637), and cytosol (528), with only 155 localized to the chloroplast (Figure 6C). Conversely, a large fraction of down‐regulated genes (580) was predicted to function in the chloroplast (Figure 6D), aligning with the observed chloroplast impairments in *xan‐h.chli‐1* under cold stress. Enriched GO terms for up‐regulated genes included stress‐related categories like “response to oxygen‐containing compounds” (131), “response to cold” (30), and “programmed cell death” (23) and membrane re‐organization (Figure 6E). Down‐regulated terms were primarily associated with chloroplast organization, photosynthesis and tetrapyrrole biosynthesis including genes encoding all four Mg‐chelatase subunits (*HvCHLI, HvCHLD, HvCHLH*, and *HvGun4*; Figure 6F). Another large fraction of the enriched down‐regulated GO categories was involved in the cell wall formation and shaping like “cell wall organization” (75), “polysaccharide metabolic process” (86), and “lignin metabolic process” (12).

In addition, the *Xan‐h* 4°C vs. *Xan‐h* 22°C comparison identified 194 unique up‐regulated genes localized primarily to the nucleus, and a comparable number resides in the chloroplast (Figure S11A,B). In contrast, the *xan‐h.chli‐1* 4°C vs. *xan‐h.chli‐1* 22°C comparison (Figure S11C,D) reflects the *xan‐h.chli‐1* 4°C vs. *Xan‐h* 4°C pattern, with most up‐regulated genes active in the nucleus (670) and only 81 in the chloroplast (Figure S11C–F).

These findings indicate that the impaired chloroplast development due to the marked reduction of chlorophyll biosynthesis under cold conditions leads to a general down‐regulation of chloroplast‐related genes. The associated reduction in chloroplast activities is most probably responsible for the down‐regulation of genes involved in polysaccharide‐, cell wall‐, lignin‐, pectin‐, and hemicellulose‐metabolism (Hori et al. 2020; Le Gall et al. 2015) and for triggering stress‐related adaptive responses.

## 4 Discussion

Although often overlooked in breeding programs, traits associated with photosynthetic efficiency have significant potential to enhance crop productivity and resilience (Croce et al. 2024). The reduction of leaf chlorophyll levels has been proposed to boost the efficiency of solar energy conversion (ECE) and improve biomass production, facilitating light absorption throughout the canopy (Cutolo et al. 2023). However, not all pale‐green mutants exhibit comparable behavior under field conditions, as optimal chlorophyll levels vary across environments and latitudes (Li et al. 2018). While a strongly reduced amount of chlorophyll is obviously difficult for the plant's metabolism to tolerate, changes in chlorophyll synthesis can trigger a wide range of pleiotropic effects (Woodson et al. 2015; Lee et al. 2003; Kim et al. 2023; Pospíšil 2016). Disruptions in the tetrapyrrole biosynthesis pathway can lead to cytotoxic accumulation of chlorophyll and heme precursors, which are highly reactive under light conditions, especially in their free forms, leading to leaf necrotic phenotypes (Woodson et al. 2015; Lee et al. 2003; Kim et al. 2023; Pospíšil 2016). This is observed when a disrupted enzymatic step of porphyrin synthesis within the chlorophyll metabolic pathway leads to an accumulation of the catalytic substrate.

In this study, we investigated the pale‐green barley mutant *xan‐h.chli‐1*, previously isolated and characterized under greenhouse and spring field conditions (Persello et al. 2024), under cold conditions. While several pale‐green mutants in Arabidopsis and barley, such as *cs/cs*, *chlorina‐f2.101*, *chlorina.111*, and *hus1*, did not exhibit cold sensitivity, the *xan‐h.chli‐1* allele showed a unique susceptibility, leading to lethality under winter field conditions, supporting the notion that the severity of the *xan‐h.chli‐1* mutation is responsible for additional physiological effects (Figure 1, Figure S1). Cold conditions intensified the chlorosis pattern along the second leaf blade of *xan‐h.chli‐1* (Figures 2, 3), amplifying the gradient of chloroplast accumulation typically observed across monocot leaf blades (Smillie et al. 1978; Pogson et al. 2015; Nelissen et al. 2016; Loudya et al. 2021). Basal (younger) regions of *xan‐h.chli‐1* leaves contained non‐photosynthetic, undifferentiated proplastid‐like organelles, characterized by simple membrane structures, large nucleoids, and carrying high levels of proplastid‐specific Nuclear Encoded RNA Polymerase (*HvSCA3*) transcripts. Interestingly, the Oxygen Evolving Complex subunit PsbO, recently proposed as a chloroplast pioneer protein in the transition from proplastids to chloroplasts (Jeran et al. 2025; Di Silvestre et al. 2025), was the only thylakoid electron transport chain subunit found to accumulate (Figure 3, Figure S4). Conversely, apical regions displayed a heterogeneous chloroplast population, including both wild‐type‐like and structurally compromised or bursting forms. These apical sections also exhibited increased expression of stress‐responsive genes (Figure 3, Figure S4).

Despite this transcriptional stress response, measurement of total ROS accumulation on whole leaves of plants grown for 10 days under cold conditions revealed lower levels in xan‐*h.chli‐1* compared to *Xan‐h*, resulting in lower leaf chlorophyll content and a lower amount of leaf chlorophyll intermediates (Figure 4). These findings are in line with the observations that mutants altered in the Mg‐chelatase activity generally show a reduction in tetrapyrrole biosynthesis, likely triggered by feedback regulatory mechanisms active in plants, aimed at reducing ALA accumulation but absent in algae (Brzezowski et al. 2016). Similar observations have also been reported in mutants and antisense RNA inactivation for *uroporphyrinogen III decarboxylase* (*UROD*), *coproporphyrinogen III oxidase* (*CPOX*), *protoporphyrinogen oxidase* (*PPOX*) and *Mg‐protoporphyrin IX monomethyl ester cyclase* (*MgPME*) genes (Mochizuki et al. 2010; Czarnecki and Grimm 2012; Brzezowski et al. 2015; Wang et al. 2022). Intriguingly, the chlorosis gradient observed throughout the *xan‐h.chli‐1* leaf blade was partially reversible. Upon returning to greenhouse growth conditions, the basal and middle sections of *xan‐h.chli‐1* leaves recovered the photosynthetic capacity and developed fully functional chloroplasts. On the contrary, photosynthetic efficiency in the apical leaf sections dramatically dropped (Figure 2), most probably as a consequence of the marked increase of chlorophyllide *a* levels in the *xan‐h.chli‐1* apical leaf section. Taken together, these leaf phenotypes indicate that proplastid‐to‐chloroplast differentiation was delayed in the mutant upon prolonged growth at 4°C. However, it was able to re‐initiate chloroplast biogenesis when the temperature was raised to control conditions. Instead, already differentiated chloroplasts carrying an increased amount of chlorophyllide *a* were damaged in cold conditions and could not recover if shifted to physiological temperatures.

The observed cold‐conditional phenotype was supported by MD simulations, employed to probe the temperature‐dependent interactions of Arg298 and Lys298 with ATP within the *Hv*CHLI binding cleft (Figure 5, Figures S5–S10). Although the analysis relied on predicted models and out‐of‐equilibrium simulations, the observations provide valuable insights into the molecular basis of temperature‐dependent functionality. The simulations revealed a high degree of conformational flexibility in the adenine ring of ATP throughout the trajectories, suggesting that the ATP binding pocket may not have reached an equilibrium state. Given that the initial ATP conformation was determined via docking, this pronounced dynamic behavior likely reflects incomplete stabilization of ATP within the binding pocket under the simulated conditions. Nevertheless, the results demonstrate that the Arg‐to‐Lys substitution at position 298 introduces temperature‐dependent interaction profiles that may underline the reduced functionality of the mutant protein at low temperatures (Figure 5). Mechanistically, MD simulations suggest that the side‐chain geometry and chemical properties of arginine enable robust ATP binding across a range of temperatures. In contrast, the lysine substitution disrupts this compatibility under cold conditions, most probably due to the reduced ability of the Lys298 side‐chain to maintain favorable contacts with the adenine ring and ribose moieties at 4°C.

To study the prolonged cold exposition effects on *xan‐h.chli‐1*, analysis of the gene networks was performed on the middle leaf section of *Xan‐h* and *xan‐h.chli‐1* plants (Figure 6, Table S2).

Due to the impaired proplastid‐to‐chloroplast differentiation rate (Figure 3), cold‐exposed *xan‐h.chli‐1* displayed a broad down‐regulation of plastid‐related genes, as indicated by large number of down‐regulated nuclear genes encoding plastid‐targeted proteins in the transcriptomic analyses (Figure 6). Gene Ontology (GO) enrichment analysis further revealed significant suppression of pathways related to tetrapyrrole biosynthesis, photosynthetic light and dark reactions, and plastid organization. It is widely documented that cold responses involve extensive metabolic and molecular reprogramming across multiple cellular pathways, including those related to carbohydrates, cell walls, lipids, amino acids, and antioxidants, to facilitate the production of cryoprotective and osmoprotective compounds. Therefore, adaptation to cold environments requires metabolically active chloroplasts (Kidokoro et al. 2022; Hori et al. 2020; Dabravolski and Isayenkov 2023; Le Gall et al. 2015; Taylor et al. 2009). The broad down‐regulation, in *xan‐h.chli‐1*, of cell wall‐related pathways, like “cell wall organization or biogenesis” (61), “pectin metabolic process” (25) and “lignin metabolic process” (12), suggest an altered cold adaptation response. Accordingly, the down‐regulation of 75 genes belonging to the GO term “response to cold” (GO:0009409) was observed, while 30 genes from the same category showed up‐regulation as possible compensatory mechanism.

On the other hand, among the few up‐regulated plastid‐localized genes in the “response to cold” GO category was *ELIP1* (Early Light‐Induced Protein 1). This gene encodes a protein that prevents excessive accumulation of free chlorophyll by inhibiting the entire chlorophyll biosynthesis pathway, including 5‐aminolevulinate synthesis and Mg‐chelatase (Hutin et al. 2003).

The reversible cold‐sensitive phenotype (chlorosis and lethality) is likely related to the inactivation of the Mg‐chelatase at 4°C, caused by the Arg‐to‐Lys substitution in *Hv*CHLI. In this scenario, the Mg‐chelatase in *xan‐h.chli‐1* cold‐exposed plants is not capable of exerting chlorophyll synthesis and supporting chloroplast differentiation. Unlike other mutants in tetrapyrrole biosynthesis regulation that accumulate photosensitizing reactive intermediates (Hou et al. 2021; Rai et al. 2025), alterations in Mg‐chelatase activity trigger a negative regulation aimed at decreasing ALA synthesis, thus reducing tetrapyrrole intermediates accumulation (Papenbrock et al. 2000; Brzezowski et al. 2016). In line with this, the entire tetrapyrrole pathway was decreased in cold‐exposed *xan‐h.chli‐1* (Figures 4 and 6). The hampered chlorophyll synthesis led to a stop of chloroplast development in *xan‐h.chli‐1*, determining the observed non‐photosynthetic chlorotic phenotype in younger tissues. Conversely, the fully mature and photosynthetically active chloroplasts, characterized by a higher content of chlorophyllide *a* in the apical leaf sections, accumulated damage leading to bursting upon prolonged cold exposition and bleaching when transferred back at 22°C (Figure 2).

## Author Contributions

Andrea Persello, Luca Tadini, Simona Masiero, and Paolo Pesaresi designed the study. Andrea Persello, Viola Torricella, Chiara Bertaso, Nina Gauri Capra, Anja Krieger‐Liszkay, and Silvan Petrac performed the molecular, biochemical, and physiological characterization of the mutants described. Andrea Persello and Lisa Rotasperti were responsible for the TEM images. Francesco Camerlengo and Giuseppe Sangiorgi organized the field trials that led to the identification of the TM2490/*xan‐h.chli‐1* mutant. Laura Rossini, David S. Horner, Nicolaj Jeran, and Andrea Persello took care of RNAseq and data analysis. Federico Ballabio, Riccardo Capelli, and Carlo Camilloni carried out the protein molecular dynamics simulations. Bernhard Grimm gave critical comments and revisions to the manuscript, and all authors helped to draft the manuscript. Paolo Pesaresi coordinated the study and wrote the final version of the manuscript. All authors gave final approval for publication and agree to be held accountable for the work performed therein.

## Conflicts of Interest

The authors declare no conflicts of interest.

## Supporting information


**Figure S1:** Weather conditions (values of light levels and air temperatures measured every 11 min) recorded in the Botanical Garden “Citt'a Studi” of the University of Milan (45°28*′*32.2*′′*N—9°14*′*05.0*′′*E) during two independent growing seasons—November 2022 to July 2023 and November 2023 to July 2024. (A, B) Light levels (in *μ*mol photons m^*−*2^ s^*−*1^) measured between November and July 2022–2023 (A) and 2023–2024 (B). (C, D) Air temperatures (*°*C) measured between November and July 2022–2023 (C) and 2023–2024 (D). The dots and arrowheads indicate the dates on which (i) the plants shown in Figure 1A were photographed and (ii) those on which field measurements of plants at the second leaf stage, sown either in winter or spring seasons (see Figure 1B), were taken, respectively.
**Figure S2:** Effects of high‐light (HL) (i) growing conditions and (ii) recovery on the photosynthetic parameters Fv/Fm, Y(II) and the leaf chlorophyll contents of mutant and control barley plants. (A) Comparison of visible phenotype (bright field illumination, BF), total chlorophyll content (SPAD) and PSII photosynthetic parameters between *xan‐h.chli‐1* and *Xan‐h* leaves under control conditions (14 days at 150 *μ*mol photons m^*−*2^ s^*−*1^). (B) Corresponding values for *Xan‐h* and *xan‐h.chli‐1* leaves grown for 14 days under high‐light conditions (1000 *μ*mol photons m^*−*2^ s^*−*1^). (C) Data obtained upon recovery from HL stress recovery (14 at 1000 *μ*mol photons m^*−*2^ s^*−*1^ and 5 days at 150 *μ*mol photons m^*−*2^ s^*−*1^). The photosynthetic Fv/Fm and Y(II) parameters are displayed in false colors; the color scale is shown below the images; violet corresponds to 1 and red to 0. Scale bar = 2 cm. Student's *t*‐test was performed to show the significance of the observed differences (*** *p <* 0.001, ** *p <* 0.01, * *p <* 0.05).
**Figure S3:** Visible phenotypes of *A. thaliana* control and mutant plants grown under induced cold stress and under optimal growth chamber conditions. (A) Visible phenotypes of *Atchli1/Atchli1* plants transformed with *Xan‐h* and *xan‐h.chli‐1* coding sequences, and the controls Col‐0 and *cs/cs* genotypes after 7 days (7 DAS) under control growth conditions (22*°*C) and after 100 days (107 DAS) of exposure to cold (4*°*C) growth conditions. (B) PSII photosynthetic parameters obtained after 7 days (7 DAS) under control conditions (22*°*C) and after 100 days (107 DAS) under cold (4*°*C) conditions. (C) Immunoblot analyses of plastid‐located Mg‐chelatase subunits (*At*CHLI, *At*CHLD, *At*GUN4) extracted from *Atchli1/Atchli1* plants that had been transformed with *Xan‐h* and *xan‐h.chli‐1* coding sequences, and the Col‐0 and *cs/cs* controls. Plants were grown for 16 days under control conditions. A portion of an SDS‐PA gel stained with Coomassie Brilliant Blue (CBB), containing the heavy chain of the ribulose bisphosphate carboxylase/oxygenase (*At*RbcL) is shown as a loading control. Scale bar = 1 cm. Student's *t*‐test was performed to show the significance of the observed differences (*** *p <* 0.001, ** *p <* 0.01, * *p <* 0.05).
**Figure S4:** Differential gene expression analysis performed on *xan‐h.chli‐1* and *Xan‐h* leaf sections under induced cold stress conditions. (A) Relative levels of marker genes known to be up‐regulated in proplastid‐containing tissues (*HvSCA3*; *HvWRKY40*), stress‐related marker genes (*HvHSFA4A*; *HvLOX2*) and ROS‐related marker genes (*HvFLOT3; HvCAT1*) was monitored by RT‐qPCR (see also Materials and methods) on RNA extracted from plants grown initially for 14 days at 22°C and then for 25 days at 4°C. (B, C) Light‐induced hydroxyl radical formation in thylakoid membranes isolated from the second leaf of *Xan‐h* and *xan‐h.chli‐1* plants exposed to cold stress (14 days at 22°C and then for 25 days at 4°C). Generation of hydroxyl radicals originating from ^•‐^O_2_/H_2_O_2_ was detected by spin trapping with 4‐POBN as a spin trap. Typical EPR spectra of the 4‐POBN/*α*‐hydroxyethyl adduct are shown (B). (C) Differences in EPR signal sizes reported as average values (*n* = 8, 2 thylakoid preparations). Letters indicate ANOVA classification based on Tukey's HSD test (Tukey's *p*‐value < 0.05).
F**igure S5**. Dimer of wild‐type and mutant (Arg298Lys) *HvCHLI* used for molecular dynamics simulations, the model of ATP^4*−*^, Arg, and Lys with CHARMM force‐field atom names. (A) Left panel: Representative hexameric ring model of wild‐type and mutant *HvCHLI*. Only the dimer containing ATP and a magnesium ion (not shown) was used, with chain A colored in light grey and chain B colored in blue. Right panel: Representative dimer model of wild‐type and mutant *Hv*CHLI. To preserve the original dimeric fold, positional restraints were applied during the simulations to the backbone of the following residues: Gly151‐Val166, Asp225‐Phe255, and Leu313‐Phe412 in chain A; Leu129‐Gly213, Val282‐Asp336, and Val379‐Phe412 in chain B. Regions with positional restraints are shown in yellow. Models are represented in cartoon form, with chain A colored in light grey and chain B colored in blue. (B) The model of ATP with the center of mass of the C4 and C5 atoms was used to approximate the center of the adenine group of ATP. (C) The model of Arg298 in the dimer of the wild‐type version of *Hv*CHLI. (D) The model of Lys298 in the dimer of the mutant version of *Hv*CHLI. All models are represented in stick forms, with carbon atoms in light grey and nitrogen atoms in blue; hydrogen atoms are not shown. The atoms involved in the analysis are indicated and labeled according to the CHARMM force‐field conventions.
**Figure S6:** Analysis of the distance between ATP and the Arg298 side chain in the wild‐type system at 4*°*C (277.15 K), which was used to evaluate possible hydrogen‐bond formation during MD simulations. (A) Distances between the NE (light blue), NH1 (blue), and NH2 (purple) atoms of Arg298 and the N3 atom of the ATP adenine ring. Each plot (top, middle and bottom) corresponds to an independent 2‐*μ*s MD simulation. Each frame is printed with a stride of 100 ps. The grey shaded area, bounded by the dashed lines at 2.2 Å and 3.8 Å, indicates the range where hydrogen bond formation is possible. (B) Distances between the NE (light blue), NH1 (blue), and NH2 (purple) atoms of Arg298 and the O4′ atom of the ATP ribose ring. Each plot (top, middle and bottom) corresponds to an independent 2 *μ*s MD simulation. Each frame is printed with a stride of 100 ps. The grey shaded area, bounded by the dashed lines at 2.2 and 3.8 Å, indicates the range where hydrogen bond formation is possible.
**Figure S7:** Distance analysis between ATP and the Arg298 side chain in the wt system at 22*°*C (295.15 K) to evaluate possible hydrogen bond formation during MD simulations. (A) Distances between the NE (light yellow), NH1 (brown), and NH2 (orange) atoms of Arg298 and the N3 atom of the ATP adenine ring. Each plot (top, middle and bottom) corresponds to an independent 2‐*μ*s MD simulation. Each frame is printed with a stride of 100 ps. The grey shaded area, bounded by the dashed lines at 2.2 and 3.8 Å, indicates the range where hydrogen bond formation is possible. (B) Distances between the NE (light yellow), NH1 (brown), and NH2 (orange) atoms of Arg298 and the O4′ atom of the ATP ribose ring. Each plot (top, middle and bottom) corresponds to an independent 2‐*μ*s MD simulation. Each frame is printed with a stride of 100 ps. The grey shaded area, bounded by the dashed lines at 2.2 and 3.8 Å, indicates the range where hydrogen bond formation is possible.
**Figure S8:** Distance analysis between ATP and the Lys298 side chain in the mutant (Arg298Lys) system at 4*°*C (277.15 K) to evaluate possible hydrogen bond formation during MD simulations. (A) The distances between the NZ atom of Lys298 and the N3 atom of the ATP adenine ring are shown in light blue. Each plot (top, middle and bottom) corresponds to an independent 2‐*μ*s MD simulation. Each frame is printed with a stride of 100 ps. The grey shaded area, bounded by the dashed lines at 2.2 and 3.8 Å, indicates the range where hydrogen bond formation is possible. (B) The distances between the NZ atom of Lys298 and the O4′ atom of the ATP ribose ring are shown in light blue. Each plot (top, middle and bottom) corresponds to an independent 2‐*μ*s MD simulation. Each frame is printed with a stride of 100 ps. The grey shaded area, bounded by the dashed lines at 2.2 and 3.8 Å, indicates the range where hydrogen bond formation is possible.
**Figure S9:** Distance analysis between ATP and the Lys298 side chain in the mutant (Arg298Lys) system at 22*°*C (295.15 K) to evaluate possible hydrogen bond formation during MD simulations. (A) The distances between the NZ atom of Lys298 and the N3 atom of the ATP adenine ring are shown in orange. Each plot (top, middle and bottom) corresponds to an independent 2‐*μ*s MD simulation. Each frame is printed with a stride of 100 ps. The grey shaded area, bounded by the dashed lines at 2.2 and 3.8 Å, indicates the range where hydrogen bond formation is possible. (B) The distances between the NZ atom of Lys298 and the O4′ atom of the ATP ribose ring are shown in orange. Each plot (top, middle and bottom) corresponds to an independent 2 *μ*s MD simulation. Each frame is printed with a stride of 100 ps. The grey shaded area, bounded by the dashed lines at 2.2 and 3.8 Å, indicates the range where hydrogen bond formation is possible.
**Figure S10:** Distance analysis between ATP and the Arg298/Lys298 side chain at 4*°*C (277.15 K) and 22*°*C (295.15 K) to evaluate possible cation‐*π* interactions during MD simulations. (A) Distances between the CZ atom of Arg298 and the center of mass of atoms C4 and C5 of the ATP adenine group at 4*°*C (277.15 K) and at 22*°*C (295.15 K). Each panel (top, middle and bottom) corresponds to an independent 2 *μ*s MD simulation at 4*°*C (light blue) and at 22*°*C (orange). Each frame is printed with a stride of 100 ps. The grey‐shaded area, fading to 6 Å, indicates the range where cation‐*π* interactions are possible. (B) Distances between the NZ atom of Lys298 and the center of mass of atoms C4 and C5 of the ATP adenine group at 4°C (277.15 K) and at 22°C (295.15 K). Each panel (top, middle and bottom) corresponds to an independent 2 μs MD simulation at 4°C (light blue) and at 22°C (orange). Each frame is printed with a stride of 100 ps. The grey‐shaded area, fading to 6 Å, indicates the range where cation‐*π* interactions are possible.
**Figure S11:** Transcriptomics and gene ontology (GO) enrichment analyses of *Xan‐h* and *xan‐h.chli‐1* middle leaf sections under induced cold stress and control conditions. (A) Volcano plot showing the differentially expressed genes (DEGs) in the middle leaf section of *Xan‐h* plants under induced cold stress conditions compared to *Xan‐h* under greenhouse conditions (*Xan‐h* 4°C vs. *Xan‐h* 22°C) after filtering (padj < 0.05 and logFC > |0.5|). (B) Predicted subcellular localization of the up‐regulated barley *Xan‐h* 4°C versus *Xan‐h* 22°C DEGs homologs in *A. thaliana*. Only the genes up‐regulated specifically in the *Xan‐h* 4°C versus *Xan‐h* 22°C comparison were selected. (C) Volcano plot showing the differentially expressed genes (DEGs) in the middle leaf section of *xan‐h.chli‐1* plants under induced cold stress conditions compared to *xan‐h.chli‐1* under greenhouse conditions (*xan‐h.chli‐1* 4°C vs. *xan‐h.chli‐1* 22°C) after filtering (padj < 0.05 and logFC > |0.5|). (D) Predicted subcellular localization of the up‐regulated barley *xan‐h.chli‐1* 4°C vs. *xan‐h.chli‐1* 22°C DEGs homologs in *A. thaliana*. Only the genes up‐regulated specifically in the *xan‐h.chli‐1* 4°C vs. *xan‐h.chli‐1* 22°C comparison were selected. (E, F) Enriched (*p* < 0.01, no of genes > 10) biological process GO terms for the up‐regulated DEGs in the (*Xan‐h* 4°C vs. *Xan‐h* 22°C; E) and *xan‐h.chli‐1* 4°C vs. *xan‐h.chli‐1* 22°C (F) comparisons. A set of representative terms is shown.

**Table S1:** List of DNA primers and plant mutant lines used in this work.

**Table S2:** Differentially expressed genes in the *xan‐h.chli‐1* under induced cold stress conditions vs. *Xan‐h* under induced cold stress conditions (*xan‐h.chli‐1* 4°C vs. *Xan‐h* 4°C). GO analysis and differentially expressed genes belonging to the GO term “response to cold” (GO:0009409) in the *xan‐h.chli‐1* 4°C versus *Xan‐h* 4°C. The table indicates both the MorexV3 gene IDs (morexV3_id) and the *A. thaliana* homologous genes (plaza_arath_ortho_id).

## Data Availability

The raw RNA‐Seq data have been deposited in the NCBI data repository (https://submit.ncbi. nlm.nih.gov/subs/bioproject/) under the bioproject identifier PRJNA1052990. The wild‐type and Arg298Lys models of the *Hv*CHLI subunit are available at ModelArchive under the accession codes ma‐xoqwu and ma‐tvik6, respectively. Relevant molecular dynamics input files and trajectories are available on Zenodo (DOI: https://doi.org/10.5281/zenodo.14680350).

## References

[ppl70434-bib-0001] Bassi, R. , and D. Simpson . 1987. “Chlorophyll‐Protein Complexes of Barley Photosystem.” European Journal of Biochemistry 163, no. 2: 221–230. 3545828 10.1111/j.1432-1033.1987.tb10791.x

[ppl70434-bib-0002] Beard, H. , A. Cholleti , D. Pearlman , W. Sherman , and K. A. Loving . 2013. “Applying Physics‐Based Scoring to Calculate Free Energies of Binding for Single Amino Acid Mutations in Protein‐Protein Complexes.” PLoS One 8, no. 12: e82849. 24340062 10.1371/journal.pone.0082849 PMC3858304

[ppl70434-bib-0003] Bell, E. , R. A. Creelman , and J. E. Mullet . 1995. “A Chloroplast Lipoxygenase Is Required for Wound‐Induced Jasmonic Acid Accumulation in Arabidopsis.” Proceedings of the National Academy of Sciences 92, no. 19: 8675–8679. 10.1073/pnas.92.19.8675 PMC41029 7567995

[ppl70434-bib-0004] Berman, H. M. , J. Westbrook , Z. Feng , et al. 2000. “The Protein Data Bank.” Nucleic Acids Research 28, no. 1: 235–242. 10592235 10.1093/nar/28.1.235 PMC102472

[ppl70434-bib-0005] Bernetti, M. , and G. Bussi . 2020. “Pressure Control Using Stochastic Cell Rescaling.” Journal of Chemical Physics 153: 114107. 32962386 10.1063/5.0020514

[ppl70434-bib-0006] Bochtler, M. , C. Hartmann , H. K. Song , G. P. Bourenkov , H. D. Bartunik , and R. Huber . 2000. “The Structures of HslU and the ATP‐Dependent Protease HslU–HslV.” Nature 403, no. 6771: 800–805. 10693812 10.1038/35001629

[ppl70434-bib-0007] Bossmann, B. , J. Knoetzel , and S. Jansson . 1997. “Screening of Chlorina Mutants of Barley (*Hordeum Vulgare* L.) With Antibodies Against Light‐Harvesting Proteins of PS I and PS II: Absence of Specific Antenna Proteins.” Photosynthesis Research 52, no. 2: 127–136.

[ppl70434-bib-0008] Brooks, B. R. , C. L. Brooks III , A. D. Mackerell Jr. , et al. 2009. “CHARMM: The Biomolecular Simulation Program.” Journal of Computational Chemistry 30, no. 10: 1545–1614. 19444816 10.1002/jcc.21287 PMC2810661

[ppl70434-bib-0009] Brzezowski, P. , A. S. Richter , and B. Grimm . 2015. “Regulation and Function of Tetrapyrrole Biosynthesis in Plants and Algae.” Biochimica et Biophysica Acta (BBA)–Bioenergetics 1847, no. 9: 968–985. 25979235 10.1016/j.bbabio.2015.05.007

[ppl70434-bib-0010] Brzezowski, P. , M. N. Sharifi , R. M. Dent , M. K. Morhard , K. K. Niyogi , and B. Grimm . 2016. “Mg Chelatase in Chlorophyll Synthesis and Retrograde Signaling in *Chlamydomonas reinhardtii* : CHLI2 Cannot Substitute for CHLI1.” Journal of Experimental Botany 67, no. 13: 3925–3938. 26809558 10.1093/jxb/erw004 PMC4915523

[ppl70434-bib-0011] Bussi, G. , D. Donadio , and M. Parrinello . 2007. “Canonical Sampling Through Velocity Rescaling.” Journal of Chemical Physics 126: 014101. 17212484 10.1063/1.2408420

[ppl70434-bib-0012] Campbell, B. W. , D. Mani , S. J. Curtin , et al. 2015. “Identical Substitutions in Magnesium Chelatase Paralogs Result in Chlorophyll‐Deficient Soybean Mutants.” G3: Genes, Genomes, Genetics 5, no. 1: 123–131. 10.1534/g3.114.015255 PMC4291463 25452420

[ppl70434-bib-0013] Canham, C. D. , A. C. Finzi , S. W. Pacala , and D. H. Burbank . 1994. “Causes and Consequences of Resource Heterogeneity in Forests: Interspecific Variation in Light Transmission by Canopy Trees.” Canadian Journal of Forest Research 24, no. 2: 337–349.

[ppl70434-bib-0014] Croce, R. , E. Carmo‐Silva , Y. B. Cho , et al. 2024. “Perspectives on Improving Photosynthesis to Increase Crop Yield.” Plant Cell 36, no. 10: 3944–3973. 38701340 10.1093/plcell/koae132 PMC11449117

[ppl70434-bib-0015] Cutolo, E. A. , Z. Guardini , L. Dall'Osto , and R. Bassi . 2023. “A Paler Shade of Green: Engineering Cellular Chlorophyll Content to Enhance Photosynthesis in Crowded Environments.” New Phytologist 239, no. 5: 1567–1583. 37282663 10.1111/nph.19064

[ppl70434-bib-0016] Czarnecki, O. , and B. Grimm . 2012. “Post‐Translational Control of Tetrapyrrole Biosynthesis in Plants, Algae, and Cyanobacteria.” Journal of Experimental Botany 63, no. 4: 1675–1687. 22231500 10.1093/jxb/err437

[ppl70434-bib-0017] Dabravolski, S. A. , and S. V. Isayenkov . 2023. “The Regulation of Plant Cell Wall Organisation Under Salt Stress.” Frontiers in Plant Science 14: 1118313. 36968390 10.3389/fpls.2023.1118313 PMC10036381

[ppl70434-bib-0018] Di Silvestre, D. , N. Jeran , G. Domingo , et al. 2025. “A Holistic Investigation of Arabidopsis Proteomes Altered in Chloroplast Biogenesis and Retrograde Signalling Identifies PsbO as a Key Regulator of Chloroplast Quality Control.” Plant, Cell & Environment 48: 6373–6396. 10.1111/pce.15611 PMC12223713 40366233

[ppl70434-bib-0019] Emanuel, C. , A. Weihe , A. Graner , W. R. Hess , and T. Börner . 2004. “Chloroplast Development Affects Expression of Phage‐Type RNA Polymerases in Barley Leaves.” Plant Journal 38, no. 3: 460–472. 10.1111/j.0960-7412.2004.02060.x 15086795

[ppl70434-bib-0020] Evans, R. , M. O'Neill , A. Pritzel , et al. 2022. “Protein Coomplex Prediction With AlphaFold‐Multimer.” bioRxiv. 10.1101/2021.10.04.463034.

[ppl70434-bib-0021] Falbel, T. G. , J. B. Meehl , and L. A. Staehelin . 1996. “Severity of Mutant Phenotype in a Series of Chlorophyll‐Deficient Wheat Mutants Depends on Light Intensity and the Severity of the Block in Chlorophyll Synthesis.” Plant Physiology 112, no. 2: 821–832. 8883392 10.1104/pp.112.2.821 PMC158007

[ppl70434-bib-0022] Fernández, A. P. , and Å. Strand . 2008. “Retrograde Signaling and Plant Stress: Plastid Signals Initiate Cellular Stress Responses.” Current Opinion in Plant Biology 11, no. 5: 509–513. 18639482 10.1016/j.pbi.2008.06.002

[ppl70434-bib-0023] Ferroni, L. , M. Živčák , O. Sytar , et al. 2020. “Chlorophyll‐Depleted Wheat Mutants Are Disturbed in Photosynthetic Electron Flow Regulation but Can Retain an Acclimation Ability to a Fluctuating Light Regime.” Environmental and Experimental Botany 178: 104156.

[ppl70434-bib-0024] Friesner, R. A. , R. B. Murphy , M. P. Repasky , et al. 2006. “Extra Precision Glide: Docking and Scoring Incorporating a Model of Hydrophobic Enclosure for Protein‐Ligand Complexes.” Journal of Medicinal Chemistry 49, no. 21: 6177–6196. 17034125 10.1021/jm051256o

[ppl70434-bib-0025] Harris, C. R. , K. J. Millman , S. J. van der Walt , et al. 2020. “Array Programming With NumPy.” Nature 585: 357–362. 32939066 10.1038/s41586-020-2649-2 PMC7759461

[ppl70434-bib-0026] Hess, B. , H. Bekker , H. J. C. Berendsen , and J. G. E. M. Fraaije . 1997. “LINCS: A Linear Constraint Solver for Molecular Simulations.” Journal of Computational Chemistry 18, no. 12: 1463–1472.

[ppl70434-bib-0027] Hori, C. , X. Yu , J. C. Mortimer , et al. 2020. “Impact of Abiotic Stress on the Regulation of Cell Wall Biosynthesis in *Populus trichocarpa* .” Plant Biotechnology 37, no. 3: 273–283. 33088190 10.5511/plantbiotechnology.20.0326a PMC7557660

[ppl70434-bib-0028] Hou, Z. , X. Pang , B. Hedtke , and B. Grimm . 2021. “In Vivo Functional Analysis of the Structural Domains of FLUORESCENT (FLU).” Plant Journal 107, no. 2: 360–376. 10.1111/tpj.15293 33901334

[ppl70434-bib-0029] Hricová, A. , V. Quesada , and J. L. Micol . 2006. “The SCABRA3 Nuclear Gene Encodes the Plastid RpoTp RNA Polymerase, Which Is Required for Chloroplast Biogenesis and Mesophyll Cell Proliferation in Arabidopsis.” Plant Physiology 141, no. 3: 942–956. 16698900 10.1104/pp.106.080069 PMC1489898

[ppl70434-bib-0030] Humphrey, W. , A. Dalke , and K. Schulten . 1996. “VMD: Visual Molecular Dynamics.” Journal of Molecular Graphics 14, no. 1: 33–38. 8744570 10.1016/0263-7855(96)00018-5

[ppl70434-bib-0031] Hunter, J. D. 2007. “Matplotlib: A 2D Graphics Environment.” Computing in Science & Engineering 9, no. 3: 90–95.

[ppl70434-bib-0032] Hutin, C. , L. Nussaume , N. Moise , I. Moya , K. Kloppstech , and M. Havaux . 2003. “Early Light‐Induced Proteins Protect Arabidopsis From Photooxidative Stress.” Proceedings of the National Academy of Sciences 100, no. 8: 4921–4926. 10.1073/pnas.0736939100 PMC153656 12676998

[ppl70434-bib-0033] Ihnatowicz, A. , P. Pesaresi , C. Varotto , et al. 2004. “Mutants for Photosystem I Subunit D of *Arabidopsis thaliana* : Effects on Photosynthesis, Photosystem I Stability and Expression of Nuclear Genes for Chloroplast Functions.” Plant Journal 37, no. 6: 839–852. 10.1111/j.1365-313x.2004.02011.x 14996217

[ppl70434-bib-0034] Jansson, C. , S. D. Wullschleger , U. C. Kalluri , and G. A. Tuskan . 2010. “Phytosequestration: Carbon Biosequestration by Plants and the Prospects of Genetic Engineering.” Bioscience 60, no. 9: 685–696.

[ppl70434-bib-0035] Jeran, N. , M. Mercier , P. Pesaresi , and L. Tadini . 2025. “Proteostasis and Protein Quality Control in Chloroplasts: Mechanisms and Novel Insights Related to Protein Mislocalization.” Journal of Experimental Botany: eraf182. 10.1093/jxb/eraf182. 40320717 PMC12485368

[ppl70434-bib-0036] Jo, S. , T. Kim , V. G. Iyer , and W. Im . 2008. “CHARMM‐GUI: A Web‐Based Graphical User Interface for CHARMM.” Journal of Computational Chemistry 29, no. 11: 1859–1865. 18351591 10.1002/jcc.20945

[ppl70434-bib-0037] Junková, P. , M. Daněk , D. Kocourková , et al. 2018. “Mapping of Plasma Membrane Proteins Interacting With *Arabidopsis thaliana* Flotillin 2.” Frontiers in Plant Science 9: 991. 30050548 10.3389/fpls.2018.00991 PMC6052134

[ppl70434-bib-0038] Kidokoro, S. , K. Shinozaki , and K. Yamaguchi‐Shinozaki . 2022. “Transcriptional Regulatory Network of Plant Cold‐Stress Responses.” Trends in Plant Science 27, no. 9: 922–935. 35210165 10.1016/j.tplants.2022.01.008

[ppl70434-bib-0039] Kim, S. J. , B. Q. Tran , and S. Jung . 2023. “Methyl Jasmonate‐Induced Senescence Results in Alterations in the Status of Chlorophyll Precursors and Enzymatic Antioxidants in Rice Plants.” Biochemical and Biophysical Research Communications 671: 38–45. 37295354 10.1016/j.bbrc.2023.06.006

[ppl70434-bib-0040] Kirst, H. , S. T. Gabilly , K. K. Niyogi , P. G. Lemaux , and A. Melis . 2017. “Photosynthetic Antenna Engineering to Improve Crop Yields.” Planta 245, no. 5: 1009–1020. 28188423 10.1007/s00425-017-2659-y

[ppl70434-bib-0041] Kirst, H. , Y. Shen , E. Vamvaka , et al. 2018. “Downregulation of the cpSRP43 Gene Expression Confers a Truncated Light‐Harvesting Antenna (TLA) and Enhances Biomass and Leaf‐To‐Stem Ratio in *Nicotiana tabacum* Canopies.” Planta 248, no. 1: 139–154. 29623472 10.1007/s00425-018-2889-7

[ppl70434-bib-0042] Kobayashi, K. , N. Mochizuki , N. Yoshimura , K. Motohashi , T. Hisabori , and T. Masuda . 2008. “Functional Analysis of *Arabidopsis thaliana* Isoforms of the Mg‐Chelatase CHLI Subunit.” Photochemical & Photobiological Sciences 7: 1188–1195. 18846282 10.1039/b802604c

[ppl70434-bib-0043] Kolberg, L. , U. Raudvere , I. Kuzmin , J. Vilo , and H. Peterson . 2020. “gprofiler2 – An R Package for Gene List Functional Enrichment Analysis and Namespace Conversion Toolset g:Profiler.” F1000Research 9: 709. 10.12688/f1000research.24956.2. PMC7859841 33564394

[ppl70434-bib-0044] Koncz, C. , R. Mayerhofer , Z. Koncz‐Kalman , et al. 1990. “Isolation of a Gene Encoding a Novel Chloroplast Protein by T‐DNA Tagging in *Arabidopsis thaliana* .” EMBO Journal 9, no. 5: 1337–1346. 2158442 10.1002/j.1460-2075.1990.tb08248.x PMC551817

[ppl70434-bib-0045] Krupinska, K. , S. Oetke , C. Desel , et al. 2014. “WHIRLY1 Is a Major Organizer of Chloroplast Nucleoids.” Frontiers in Plant Science 5: 432. 25237316 10.3389/fpls.2014.00432 PMC4154442

[ppl70434-bib-0046] Lake, V. , U. Olsson , R. D. Willows , and M. Hansson . 2004. “ATPase Activity of Magnesium Chelatase Subunit I Is Required to Maintain Subunit D In Vivo.” European Journal of Biochemistry 271, no. 11: 2182–2188. 15153108 10.1111/j.1432-1033.2004.04143.x

[ppl70434-bib-0047] Le Gall, H. , F. Philippe , J.‐M. Domon , F. Gillet , J. Pelloux , and C. Rayon . 2015. “Cell Wall Metabolism in Response to Abiotic Stress.” Plants 4, no. 1: 112–166. 27135320 10.3390/plants4010112 PMC4844334

[ppl70434-bib-0048] Lee, J. , X. Cheng , S. Jo , A. D. MacKerell , J. B. Klauda , and W. Im . 2016. “CHARMM‐GUI Input Generator for NAMD, GROMACS, AMBER, OpenMM, and CHARMM/OpenMM Simulations Using the CHARMM36 Additive Force Field.” Biophysical Journal 110, no. 3: 641a. 10.1021/acs.jctc.5b00935 PMC4712441 26631602

[ppl70434-bib-0049] Lee, K. P. , C. Kim , D. W. Lee , and K. Apel . 2003. “Tigrina d, Required for Regulating the Biosynthesis of Tetrapyrroles in Barley, Is an Ortholog of the FLU Gene of *Arabidopsis thaliana* .” FEBS Letters 553, no. 1: 119–124. 14550558 10.1016/s0014-5793(03)00983-9

[ppl70434-bib-0050] Li, Y. , C. Liu , J. Zhang , et al. 2018. “Variation in Leaf Chlorophyll Concentration From Tropical to Cold‐Temperate Forests: Association With Gross Primary Productivity.” Ecological Indicators 85: 383–389.

[ppl70434-bib-0051] Lin, Y. P. , Y. Y. Shen , Y. B. Shiu , Y. Y. Charng , and B. Grimm . 2022. “Chlorophyll Dephytylase 1 and Chlorophyll Synthase: A Chlorophyll Salvage Pathway for the Turnover of Photosystems I and II.” Plant Journal 111, no. 4: 979–994. 10.1111/tpj.15865 35694901

[ppl70434-bib-0052] Liu, R. , Y.‐H. Xu , S.‐C. Jiang , et al. 2013. “Light‐Harvesting Chlorophyll a/b‐Binding Proteins, Positively Involved in Abscisic Acid Signalling, Require a Transcription Repressor, WRKY40, to Balance Their Function.” Journal of Experimental Botany 64, no. 18: 5443–5456. 24078667 10.1093/jxb/ert307 PMC3871805

[ppl70434-bib-0053] Long, S. P. , A. Marshall‐Colon , and X.‐G. Zhu . 2015. “Meeting the Global Food Demand of the Future by Engineering Crop Photosynthesis and Yield Potential.” Cell 161, no. 1: 56–66. 25815985 10.1016/j.cell.2015.03.019

[ppl70434-bib-0054] Loudya, N. , P. Mishra , K. Takahagi , et al. 2021. “Cellular and Transcriptomic Analyses Reveal Two‐Staged Chloroplast Biogenesis Underpinning Photosynthesis Build‐Up in the Wheat Leaf.” Genome Biology 22, no. 1: 151. 33975629 10.1186/s13059-021-02366-3 PMC8111775

[ppl70434-bib-0055] Love, M. I. , W. Huber , and S. Anders . 2014. “Moderated Estimation of Fold Change and Dispersion for RNA‐Seq Data With DESeq2.” Genome Biology 15: 550. 25516281 10.1186/s13059-014-0550-8 PMC4302049

[ppl70434-bib-0056] Mauzerall, D. , and S. Granick . 1956. “The Occurrence and Determination of δ‐Aminolevulinic Acid and Porphobilinogen in Urine.” Journal of Biological Chemistry 219, no. 1: 435–446. 13295297

[ppl70434-bib-0057] McKinney, W. 2010. “Data Structures for Statistical Computing in Python.” In Proceedings of the 9th Python in Science Conference, vol. 445, 51–56. Austin, TX.

[ppl70434-bib-0058] Melis, A. 2009. “Solar Energy Conversion Efficiencies in Photosynthesis: Minimizing the Chlorophyll Antennae to Maximize Efficiency.” Plant Science 177, no. 4: 272–280.

[ppl70434-bib-0059] Méndez‐Gómez, M. , D. Sierra‐Cacho , E. Jiménez‐Morales , and P. Guzmán . 2024. “Modulation of Early Gene Expression Responses to Water Deprivation Stress by the E3 Ubiquitin Ligase ATL80: Implications for Retrograde Signaling Interplay.” BMC Plant Biology 24, no. 1: 180. 38459432 10.1186/s12870-024-04872-5 PMC10921668

[ppl70434-bib-0062] Mochizuki, N. , R. Tanaka , B. Grimm , et al. 2010. “The Cell Biology of Tetrapyrroles: A Life and Death Struggle.” Trends in Plant Science 15, no. 9: 488–498. 20598625 10.1016/j.tplants.2010.05.012

[ppl70434-bib-0064] Nelissen, H. , N. Gonzalez , and D. Inzé . 2016. “Leaf Growth in Dicots and Monocots: So Different Yet So Alike.” Current Opinion in Plant Biology 33: 72–76. 27344391 10.1016/j.pbi.2016.06.009

[ppl70434-bib-0066] Papenbrock, J. , H. P. Mock , R. Tanaka , E. Kruse , and B. Grimm . 2000. “Role of Magnesium Chelatase Activity in the Early Steps of the Tetrapyrrole Biosynthetic Pathway.” Plant Physiology 122, no. 4: 1161–1169. 10759511 10.1104/pp.122.4.1161 PMC58950

[ppl70434-bib-0067] Pérez‐Salamó, I. , C. Papdi , G. Rigó , et al. 2014. “The Heat Shock Factor A4A Confers Salt Tolerance and Is Regulated by Oxidative Stress and the Mitogen‐Activated Protein Kinases MPK3 and MPK6.” Plant Physiology 165, no. 1: 319–334. 24676858 10.1104/pp.114.237891 PMC4012591

[ppl70434-bib-0068] Persello, A. , L. Tadini , L. Rotasperti , et al. 2024. “A Missense Mutation in the Barley Xan‐H Gene Encoding the Mg‐Chelatase Subunit I Leads to a Viable Pale Green Line With Reduced Daily Transpiration Rate.” Plant Cell Reports 43, no. 10: 246. 39343835 10.1007/s00299-024-03328-2 PMC11439855

[ppl70434-bib-0069] Petersen, H. G. 1995. “Accuracy and Efficiency of the Particle Mesh Ewald Method.” Journal of Chemical Physics 103, no. 9: 3668–3679.

[ppl70434-bib-0070] Pogson, B. J. , D. Ganguly , and V. Albrecht‐Borth . 2015. “Insights Into Chloroplast Biogenesis and Development.” Biochimica et Biophysica Acta, Bioenergetics 1847, no. 9: 1017–1024. 10.1016/j.bbabio.2015.02.003 25667967

[ppl70434-bib-0071] Pospíšil, P. 2016. “Production of Reactive Oxygen Species by Photosystem II as a Response to Light and Temperature Stress.” Frontiers in Plant Science 7: 1950. 28082998 10.3389/fpls.2016.01950 PMC5183610

[ppl70434-bib-0072] Rai, S. , M. D. Lemke , A. M. Arias , M. F. Gómez Méndez , K. Dehesh , and J. D. Woodson . 2025. “Transcript Profiling of Plastid Ferrochelatase Two Mutants Reveals That Chloroplast Singlet Oxygen Signals Lead to Global Changes in RNA Profiles and Are Mediated by Plant U‐Box 4.” BMC Plant Biology 25, no. 1: 1–23. 40457237 10.1186/s12870-025-06703-7 PMC12131553

[ppl70434-bib-0074] Rotasperti, L. , L. Tadini , M. Chiara , et al. 2022. “The Barley Mutant Happy Under the Sun 1 (hus1): An Additional Contribution to Pale Green Crops.” Environmental and Experimental Botany 196: 104795.

[ppl70434-bib-0075] Sakowska, K. , G. Alberti , L. Genesio , et al. 2018. “Leaf and Canopy Photosynthesis of a Chlorophyll Deficient Soybean Mutant.” Plant, Cell & Environment 41, no. 6: 1427–1437. 10.1111/pce.13180 29498070

[ppl70434-bib-0077] Sastry, G. M. , M. Adzhigirey , T. Day , R. Annabhimoju , and W. Sherman . 2013. “Protein and Ligand Preparation: Parameters, Protocols, and Influence on Virtual Screening Enrichments.” Journal of Computer‐Aided Molecular Design 27: 221–234. 23579614 10.1007/s10822-013-9644-8

[ppl70434-bib-0078] Schägger, H. , and G. von Jagow . 1987. “Tricine‐Sodium Dodecyl Sulfate‐Polyacrylamide Gel Electrophoresis for the Separation of Proteins in the Range From 1 to 100 kDa.” Analytical Biochemistry 166, no. 2: 368–379. 2449095 10.1016/0003-2697(87)90587-2

[ppl70434-bib-0079] Schwede, T. , A. Sali , B. Honig , et al. 2009. “Outcome of a Workshop on Applications of Protein Models in Biomedical Research.” Structure 17, no. 2: 151–159. 19217386 10.1016/j.str.2008.12.014 PMC2739730

[ppl70434-bib-0080] Simpson, D. J. , O. Machold , G. Høyer‐Hansen , and D. von Wettstein . 1985. “Chlorina Mutants of Barley ( *Hordeum vulgare* L.).” Carlsberg Research Communications 50, no. 4: 223.

[ppl70434-bib-0081] Slattery, R. A. , and D. R. Ort . 2020. “Perspectives on Improving Light Distribution and Light Use Efficiency in Crop Canopies.” Plant Physiology 185, no. 1: 34–48. 10.1093/plphys/kiaa006 PMC8133579 33631812

[ppl70434-bib-0082] Smillie, R. M. , C. Critchley , J. M. Bain , and R. Nott . 1978. “Effect of Growth Temperature on Chloroplast Structure and Activity in Barley.” Plant Physiology 62, no. 2: 191–196. 16660484 10.1104/pp.62.2.191 PMC1092088

[ppl70434-bib-0083] Tadini, L. , C. Peracchio , A. Trotta , et al. 2020. “GUN1 Influences the Accumulation of NEP‐Dependent Transcripts and Chloroplast Protein Import in Arabidopsis Cotyledons Upon Perturbation of Chloroplast Protein Homeostasis.” Plant Journal 101, no. 5: 1198–1220. 10.1111/tpj.14585 31648387

[ppl70434-bib-0084] Talamè, V. , R. Bovina , M. C. Sanguineti , R. Tuberosa , U. Lundqvist , and S. Salvi . 2008. “TillMore, a Resource for the Discovery of Chemically Induced Mutants in Barley.” Plant Biotechnology Journal 6, no. 5: 477–485. 18422888 10.1111/j.1467-7652.2008.00341.x

[ppl70434-bib-0085] Taylor, N. L. , Y.‐F. Tan , R. P. Jacoby , and A. H. Millar . 2009. “Abiotic Environmental Stress Induced Changes in the *Arabidopsis thaliana* Chloroplast, Mitochondria and Peroxisome Proteomes.” Journal of Proteomics 72, no. 3: 367–378. 19061979 10.1016/j.jprot.2008.11.006

[ppl70434-bib-0086] Tribello, G. A. , M. Bonomi , D. Branduardi , C. Camilloni , and G. Bussi . 2014. “PLUMED 2: New Feathers for an Old Bird.” Computer Physics Communications 185, no. 2: 604–613.

[ppl70434-bib-0087] Van Aken, O. , B. Zhang , S. Law , R. Narsai , and J. Whelan . 2013. “AtWRKY40 and AtWRKY63 Modulate the Expression of Stress‐Responsive Nuclear Genes Encoding Mitochondrial and Chloroplast Proteins.” Plant Physiology 162, no. 1: 254–271. 23509177 10.1104/pp.113.215996 PMC3641207

[ppl70434-bib-0088] Van Der Spoel, D. , E. Lindahl , B. Hess , G. Groenhof , A. E. Mark , and H. J. C. Berendsen . 2005. “GROMACS: Fast, Flexible, and Free.” Journal of Computational Chemistry 26, no. 16: 1701–1718. 16211538 10.1002/jcc.20291

[ppl70434-bib-0089] Van Kempen, M. , S. S. Kim , C. Tumescheit , et al. 2024. “Fast and Accurate Protein Structure Search With Foldseek.” Nature Biotechnology 42, no. 2: 243–246. 10.1038/s41587-023-01773-0 PMC10869269 37156916

[ppl70434-bib-0090] Van Rossum, G. , and F. L. Drake . 2009. Python 3 Reference Manual. CreateSpace.

[ppl70434-bib-0091] Verwoerd, T. C. , B. M. M. Dekker , and A. Hoekema . 1989. “A Small‐Scale Procedure for the Rapid Isolation of Plant RNAs.” Nucleic Acids Research 17, no. 6: 2362. 2468132 10.1093/nar/17.6.2362 PMC317610

[ppl70434-bib-0092] Walker, B. J. , D. T. Drewry , R. A. Slattery , A. VanLoocke , Y. B. Cho , and D. R. Ort . 2017. “Chlorophyll Can Be Reduced in Crop Canopies With Little Penalty to Photosynthesis.” Plant Physiology 176, no. 2: 1215–1232. 29061904 10.1104/pp.17.01401 PMC5813550

[ppl70434-bib-0093] Wang, P. , S. Ji , and B. Grimm . 2022. “Post‐Translational Regulation of Metabolic Checkpoints in Plant Tetrapyrrole Biosynthesis.” Journal of Experimental Botany 73, no. 14: 4624–4636. 35536687 10.1093/jxb/erac203 PMC9992760

[ppl70434-bib-0094] Woodson, J. , M. Joens , A. Sinson , et al. 2015. “Ubiquitin Facilitates a Quality‐Control Pathway That Removes Damaged Chloroplasts.” Science 350, no. 6259: 450–454. 26494759 10.1126/science.aac7444 PMC4863637

[ppl70434-bib-0095] Xing, Y. , W. Jia , and J. Zhang . 2008. “AtMKK1 Mediates ABA‐Induced CAT1 Expression and H_2_O_2_ Production via AtMPK6‐Coupled Signaling in Arabidopsis.” Plant Journal 54, no. 3: 440–451. 10.1111/j.1365-313X.2008.03433.x 18248592

[ppl70434-bib-0096] Zenodo . 2013. “CERN.” https://zenodo.org.

