# Supplementary material for: A Mg‐Chelatase Subunit I Missense Mutant in Barley Exhibits a Cold‐Sensitive Phenotype Under Field Conditions
Source: Physiol Plant. 2025 Aug 5;177(4):e70434. doi: 10.1111/ppl.70434 (PMC12322876; doi:10.1111/ppl.70434)
Supplement: Supplementary file 1 — Figure S1: Weather conditions (values of light levels and air temperatures measured every 11 min) recorded in the Botanical Garden “Citt'a Studi” of the University of Milan (45°28′32.2′′N—9°14′05.0′′E) during two independent growing seasons—November 2022 to July 2023 and November 2023 to July 2024. (A, B) Light levels (in μmol photons m −2 s −1) measured between November and July 2022–2023 (A) and 2023–2024 (B). (C, D) Air temperatures (°C) measured between November and July 2022–2023 (C) and 2023–2024 (D). The dots and arrowheads indicate the dates on which (i) the plants shown in Figure 1A were photographed and (ii) those on which field measurements of plants at the second leaf stage, sown either in winter or spring seasons (see Figure 1B), were taken, respectively. Figure S2: Effects of high‐light (HL) (i) growing conditions and (ii) recovery on the photosynthetic parameters Fv/Fm, Y(II) and the leaf chlorophyll contents of mutant and control barley plants. (A) Comparison of visible phenotype (bright field illumination, BF), total chlorophyll content (SPAD) and PSII photosynthetic parameters between xan‐h.chli‐1 and Xan‐h leaves under control conditions (14 days at 150 μmol photons m −2 s −1). (B) Corresponding values for Xan‐h and xan‐h.chli‐1 leaves grown for 14 days under high‐light conditions (1000 μmol photons m −2 s −1). (C) Data obtained upon recovery from HL stress recovery (14 at 1000 μmol photons m −2 s −1 and 5 days at 150 μmol photons m −2 s −1). The photosynthetic Fv/Fm and Y(II) parameters are displayed in false colors; the color scale is shown below the images; violet corresponds to 1 and red to 0. Scale bar = 2 cm. Student's t‐test was performed to show the significance of the observed differences (*** p < 0.001, ** p < 0.01, * p < 0.05). Figure S3: Visible phenotypes of A. thaliana control and mutant plants grown under induced cold stress and under optimal growth chamber conditions. (A) Visible phenotypes of Atchli1/Atchli1 plants transformed wit [file PPL-177-e70434-s001.pdf]

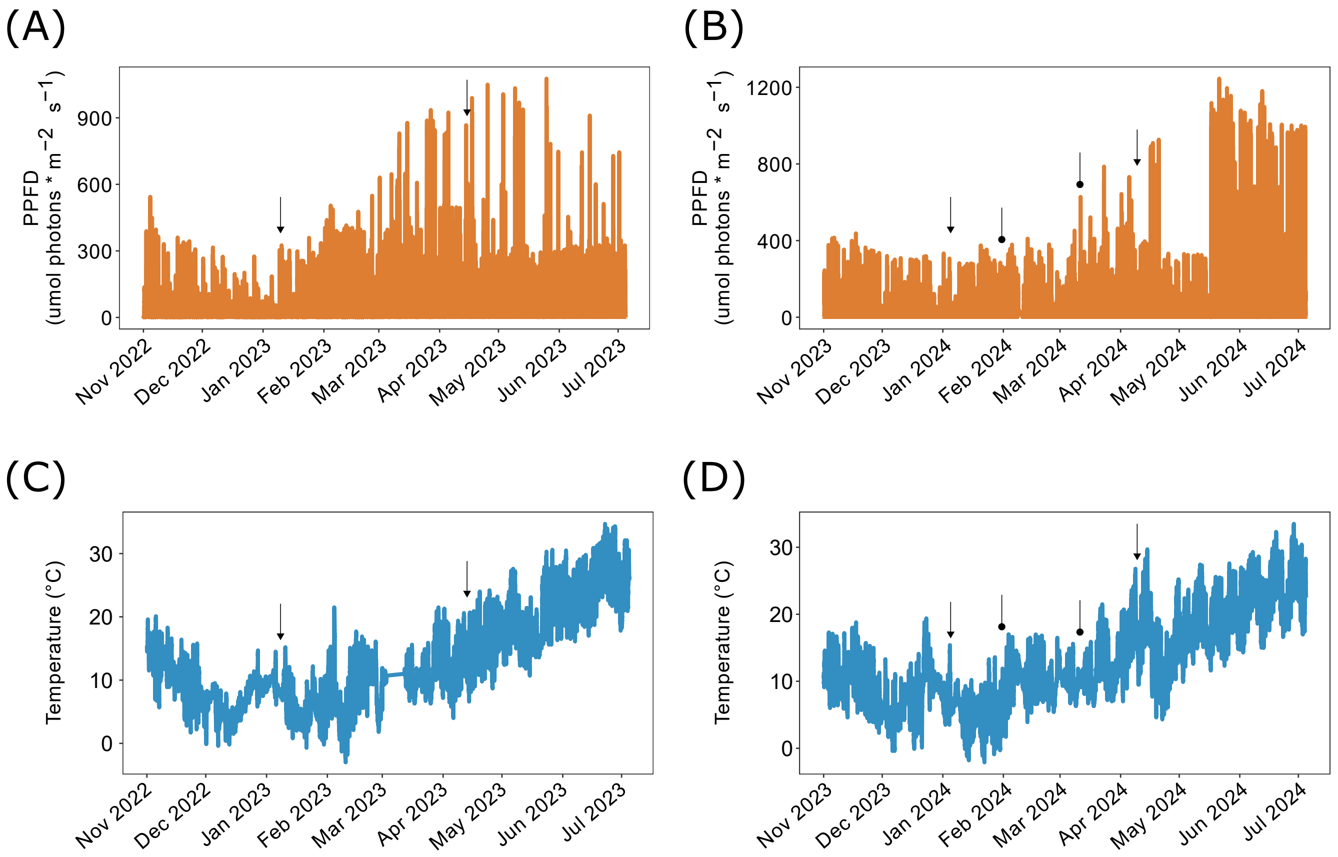
(A)
PPFD
(umol photons * m−2 s−1)
900
600
300
0
Nov 2022
Dec 2022
Jan 2023
Feb 2023
Mar 2023
Apr 2023
May 2023
Jun 2023
Jul 2023
(B)
1200
800
400
0
Nov 2023
Dec 2023
Jan 2024
Feb 2024
Mar 2024
Apr 2024
May 2024
Jun 2024
Jul 2024
(C)
Temperature (°C)
30
20
10
0
(D)

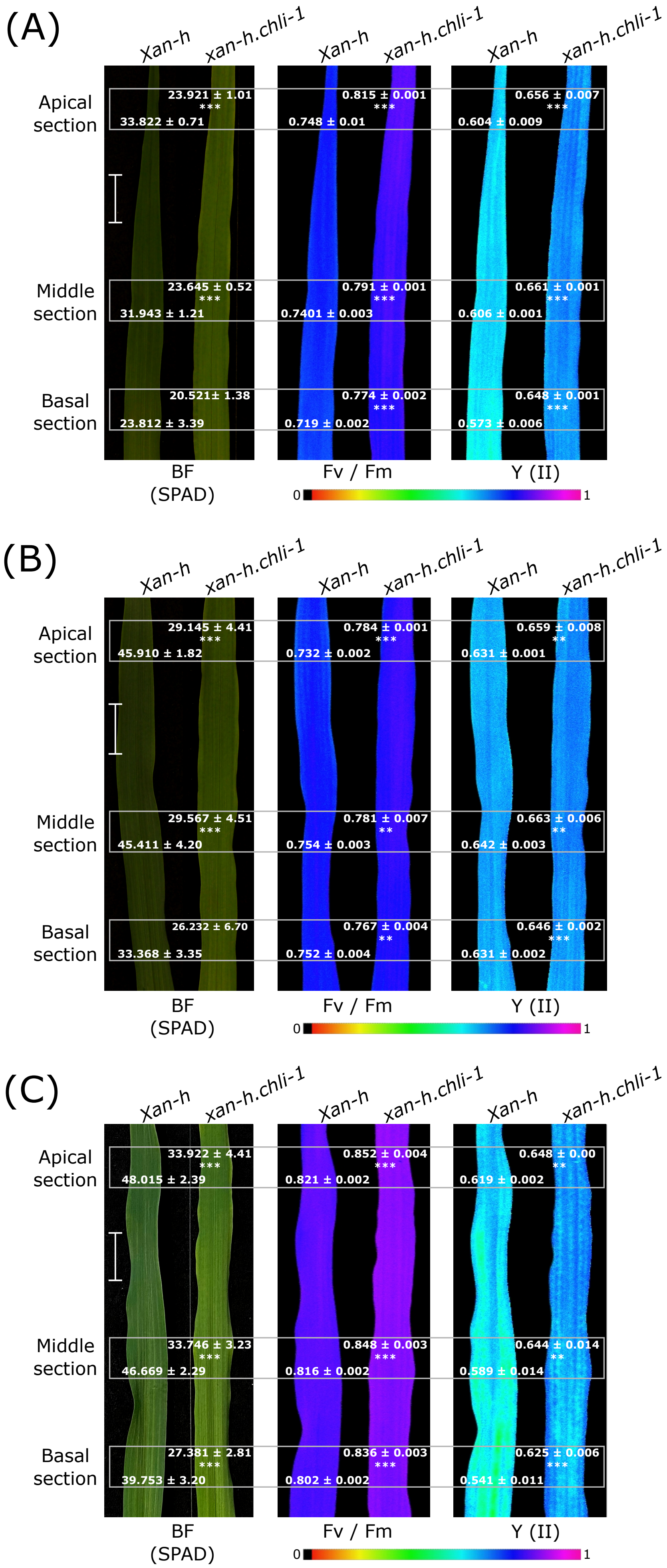

(A)
Xan-h
xan-h.chli-1
Xan-h
xan-h.chli-1
Xan-h
xan-h.chli-1
Apical section
23.921 ± 1.01
***
33.822 ± 0.71
0.815 ± 0.001
***
0.748 ± 0.01
0.656 ± 0.007
***
0.604 ± 0.009
Middle section
23.645 ± 0.52
***
31.943 ± 1.21
0.791 ± 0.001
***
0.7401 ± 0.003
0.661 ± 0.001
***
0.606 ± 0.001
Basal section
20.521± 1.38
23.812 ± 3.39
0.774 ± 0.002
***
0.719 ± 0.002
0.648 ± 0.001
***
0.573 ± 0.006
BF
(SPAD)
Fv / Fm
Y (II)
0
1
(B)
Xan-h
xan-h.chli-1
Xan-h
xan-h.chli-1
Xan-h
xan-h.chli-1
Apical section
29.145 ± 4.41
***
45.910 ± 1.82
0.784 ± 0.001
***
0.732 ± 0.002
0.659 ± 0.008
**
0.631 ± 0.001
Middle section
29.567 ± 4.51
***
45.411 ± 4.20
0.781 ± 0.007
**
0.754 ± 0.003
0.663 ± 0.006
**
0.642 ± 0.003
Basal section
26.232 ± 6.70
33.368 ± 3.35
0.767 ± 0.004
**
0.752 ± 0.004
0.646 ± 0.002
***
0.631 ± 0.002
BF
(SPAD)
Fv / Fm
Y (II)
0
1
(C)
Xan-h
xan-h.chli-1
Xan-h
xan-h.chli-1
Xan-h
xan-h.chli-1
Apical section
33.922 ± 4.41
***
48.015 ± 2.39
0.852 ± 0.004
***
0.821 ± 0.002
0.648 ± 0.00
**
0.619 ± 0.002
Middle section
33.746 ± 3.23
***
46.669 ± 2.29
0.848 ± 0.003
***
0.816 ± 0.002
0.644 ± 0.014
**
0.589 ± 0.014
Basal section
27.381 ± 2.81
***
39.753 ± 3.20
0.836 ± 0.003
***
0.802 ± 0.002
0.625 ± 0.006
***
0.541 ± 0.011
BF
(SPAD)
Fv / Fm
Y (II)
0
1

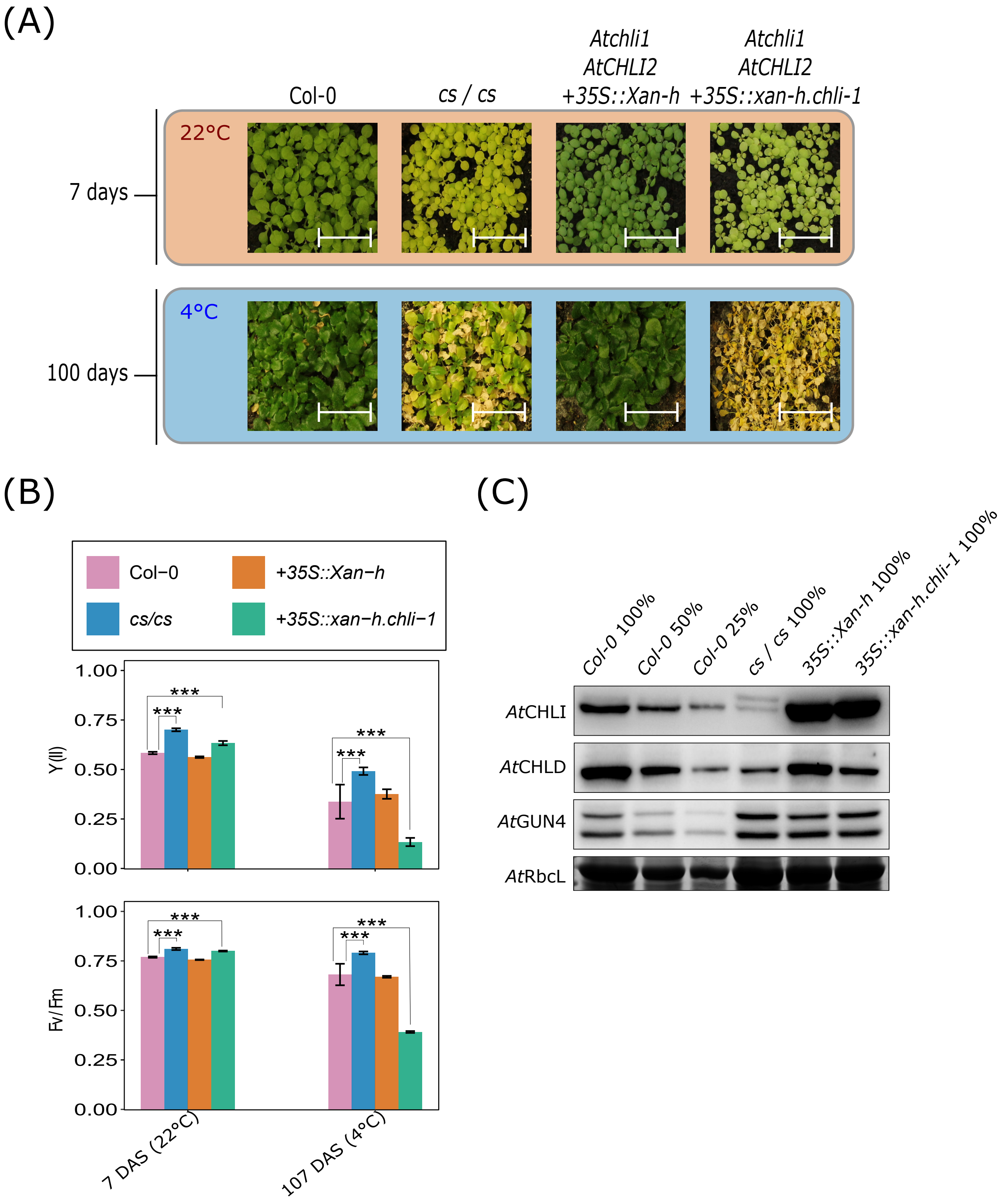

(A)
Col-0
cs / cs
Atchli1
AtCHLI2
+35S::Xan-h
Atchli1
AtCHLI2
+35S::xan-h.chli-1
7 days
22°C
100 days
4°C
(B)
Col−0
cs/cs
+35S::Xan−h
+35S::xan−h.chli−1
Y(II)
Fv/Fm
1.00
0.75
0.50
0.25
0.00
***
7 DAS (22°C)
107 DAS (4°C)
(C)
Col-0 100%
Col-0 50%
Col-0 25%
cs / cs 100%
35S::Xan-h 100%
35S::xan-h.chli-1 100%
AtCHLI
AtCHLD
AtGUN4
AtRbcL

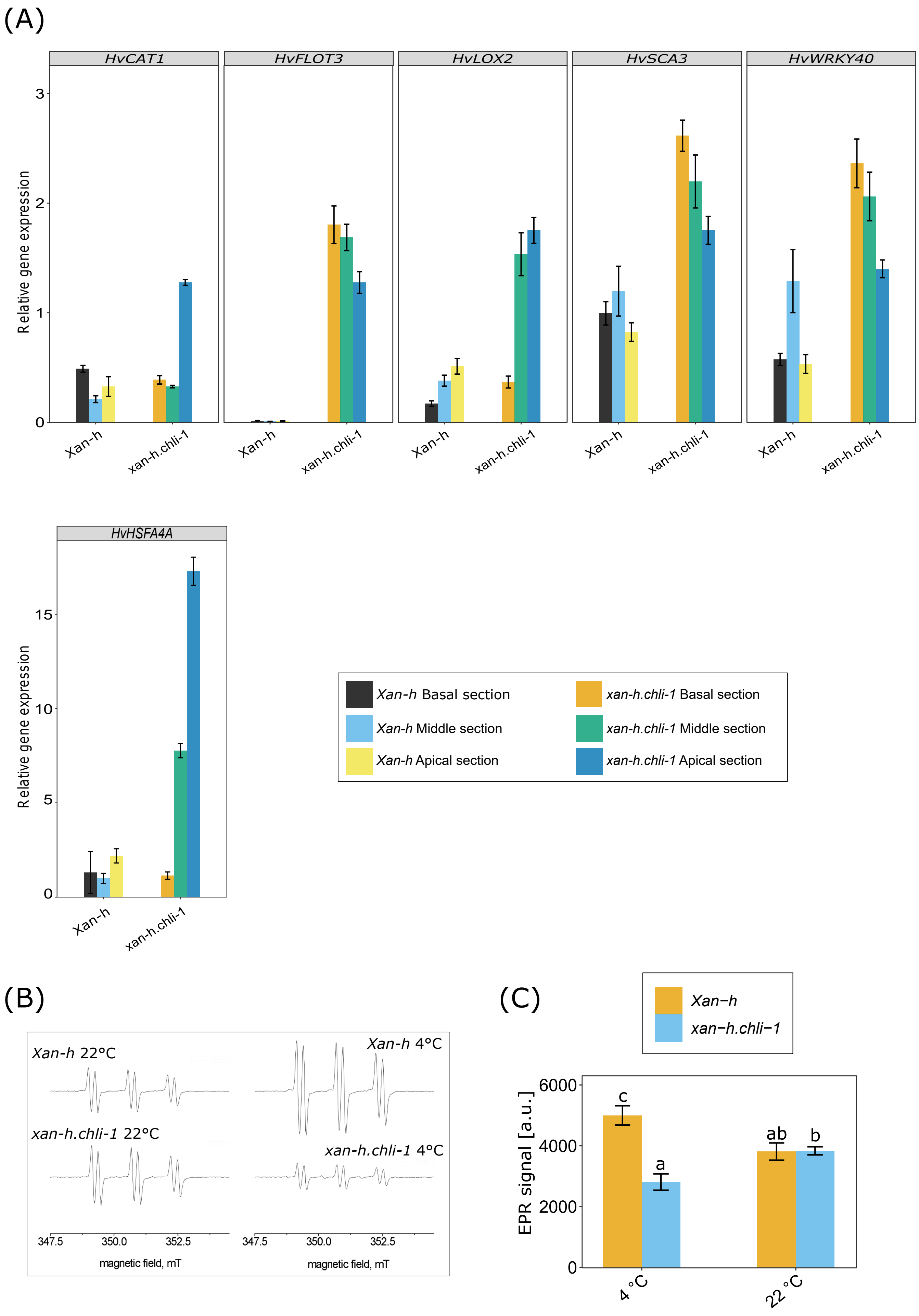

(A)
HvCAT1
HvFLOT3
HvLOX2
HvSCA3
HvWRKY40
HvHSFA4A
Relative gene expression
Xan-h
xan-h.chli-1
Xan-h Basal section
Xan-h Middle section
Xan-h Apical section
xan-h.chli-1 Basal section
xan-h.chli-1 Middle section
xan-h.chli-1 Apical section
(B)
Xan-h 22°C
Xan-h 4°C
xan-h.chli-1 22°C
xan-h.chli-1 4°C
347.5
350.0
352.5
magnetic field, mT
(C)
Xan−h
xan−h.chli−1
EPR signal [a.u.]
6000
4000
2000
0
c
a
ab
b
4 °C
22 °C

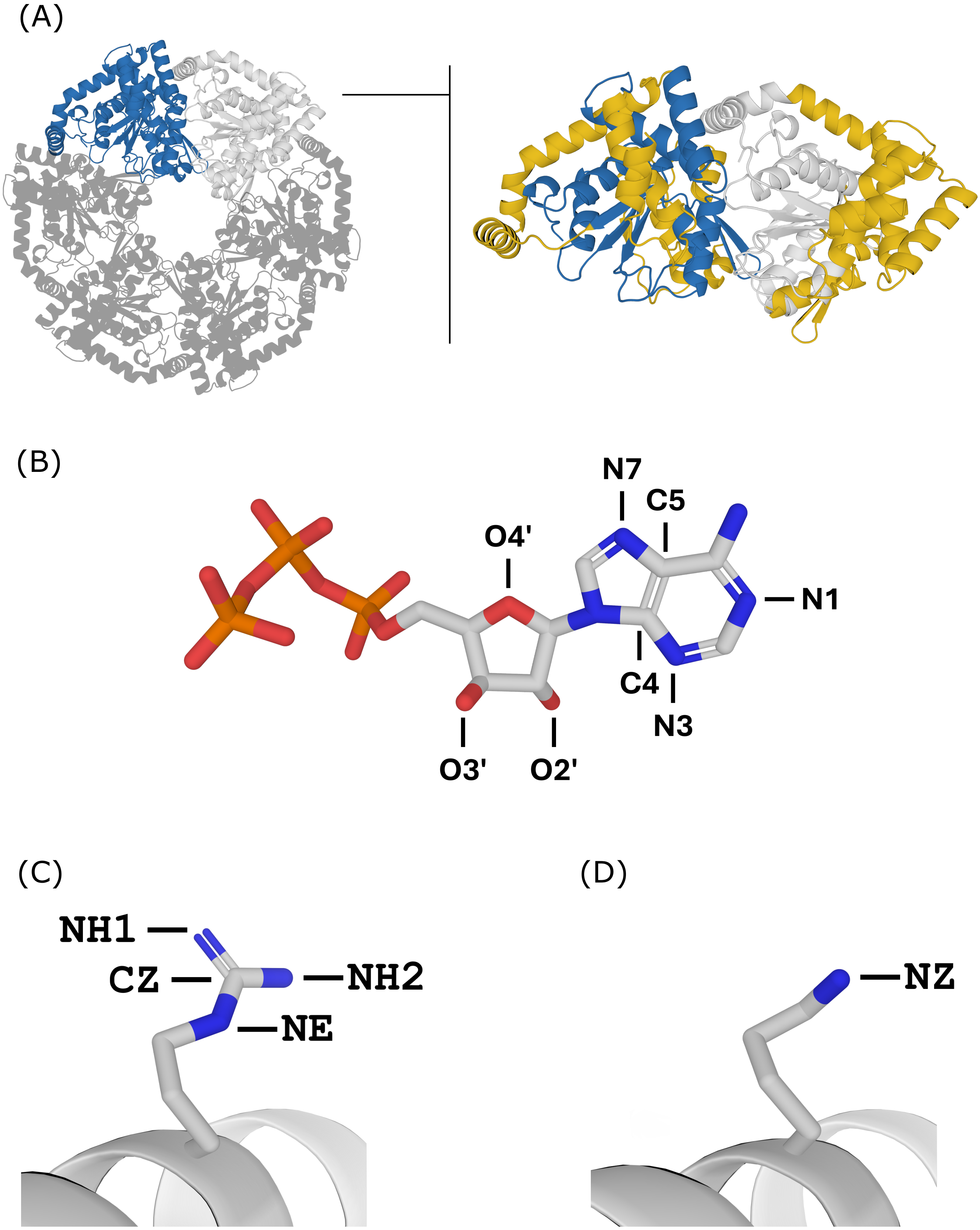

(A)
(B)
N7
C5
O4'
N1
C4
N3
O3'
O2'
(C)
(D)
NH1
CZ
NH2
NE
NZ

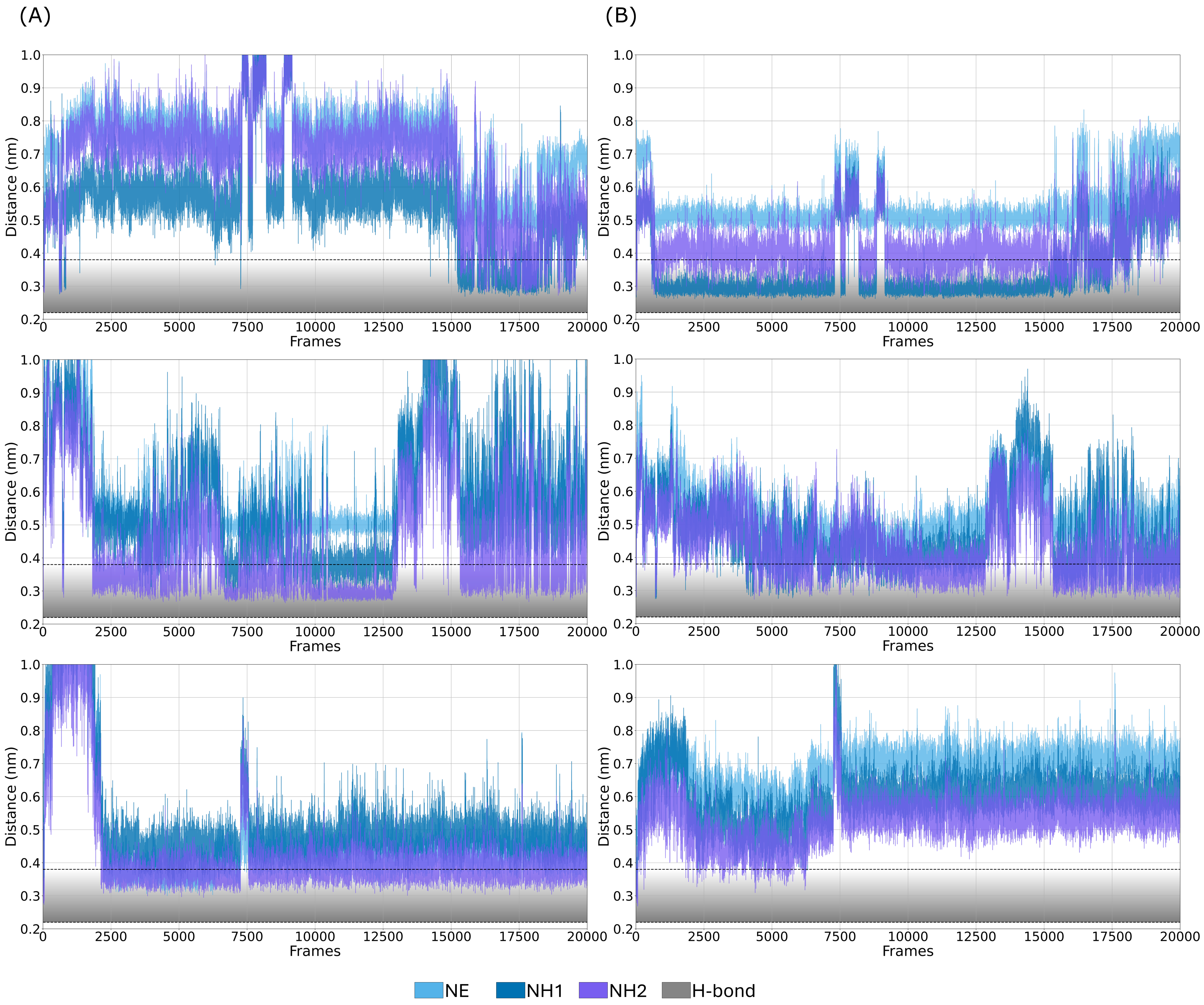
(A)
(B)
Distance (nm)
Frames
NE
NH1
NH2
H-bond

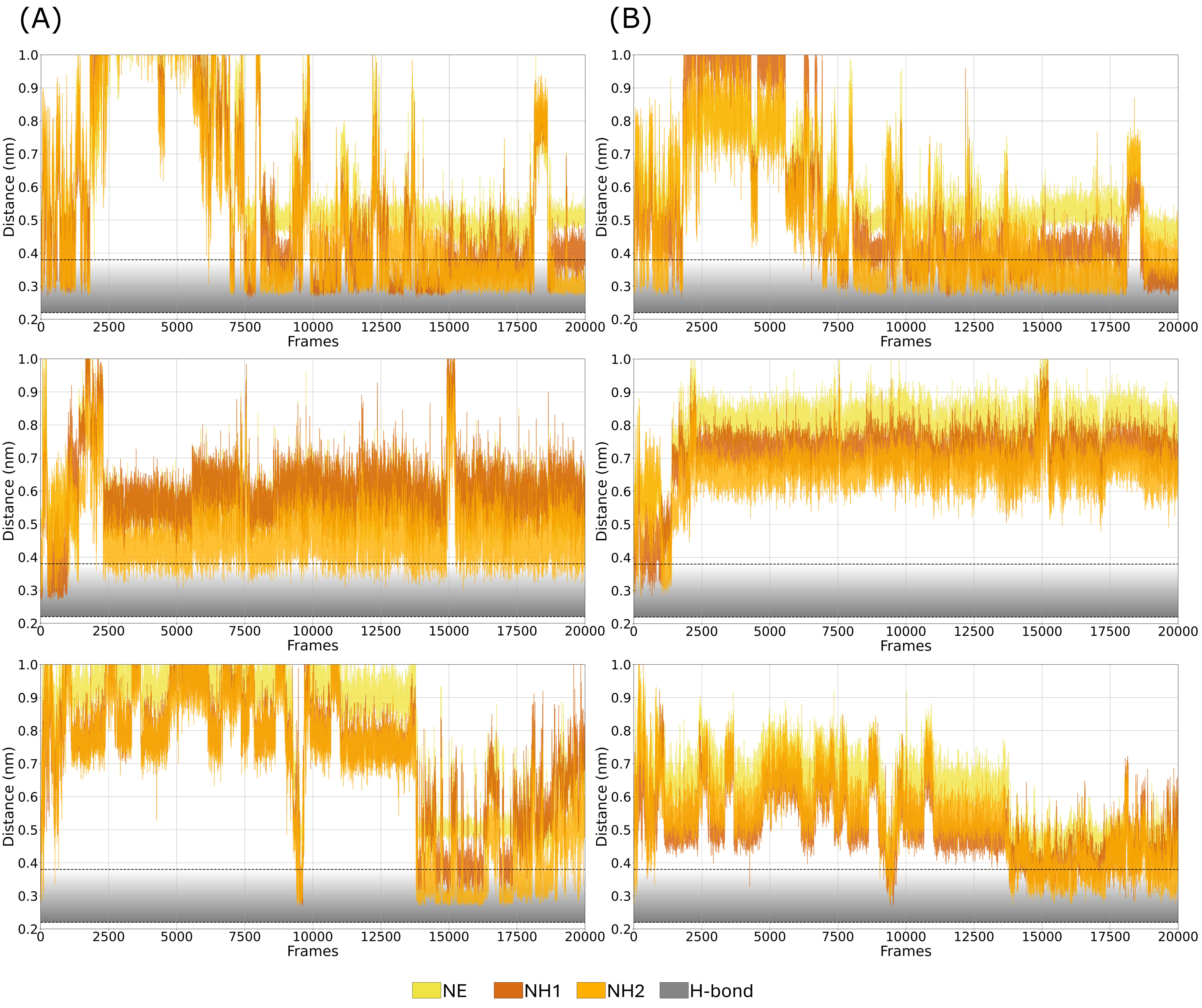
(A)
(B)
Distance (nm)
Frames
NE
NH1
NH2
H-bond

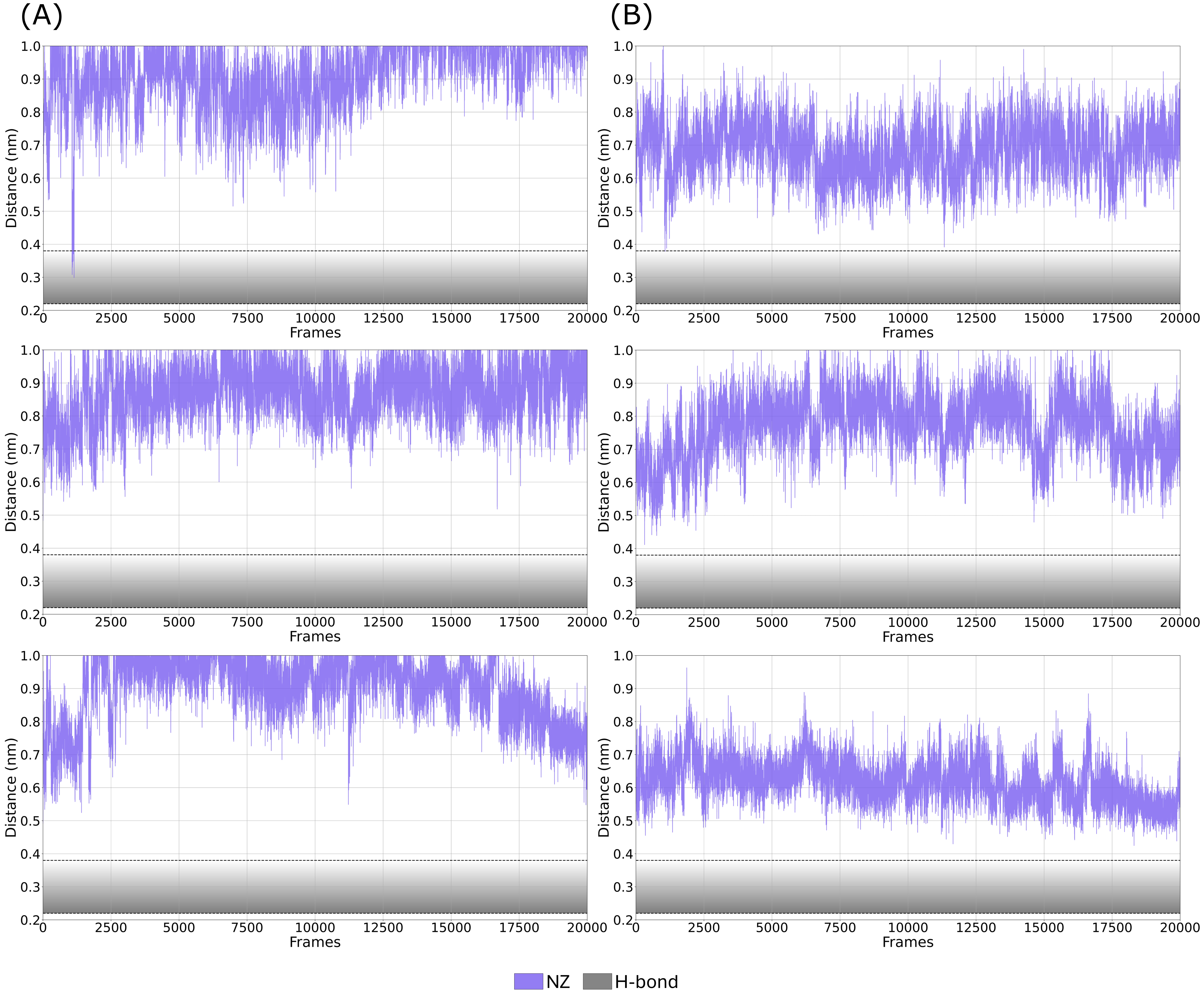

(A)
(B)
Distance (nm)
Frames
NZ
H-bond

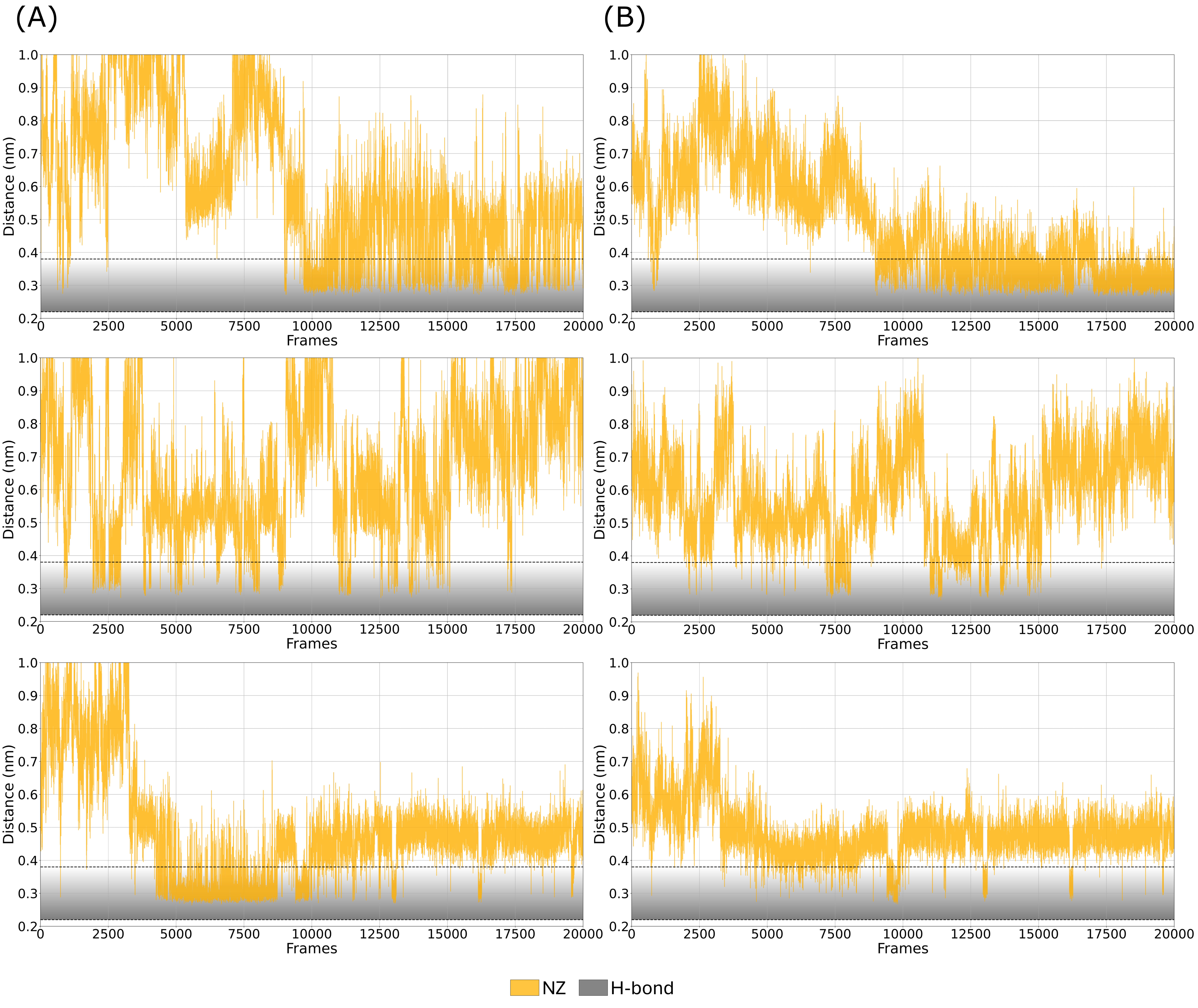
(A)
(B)
Distance (nm)
Frames
NZ
H-bond

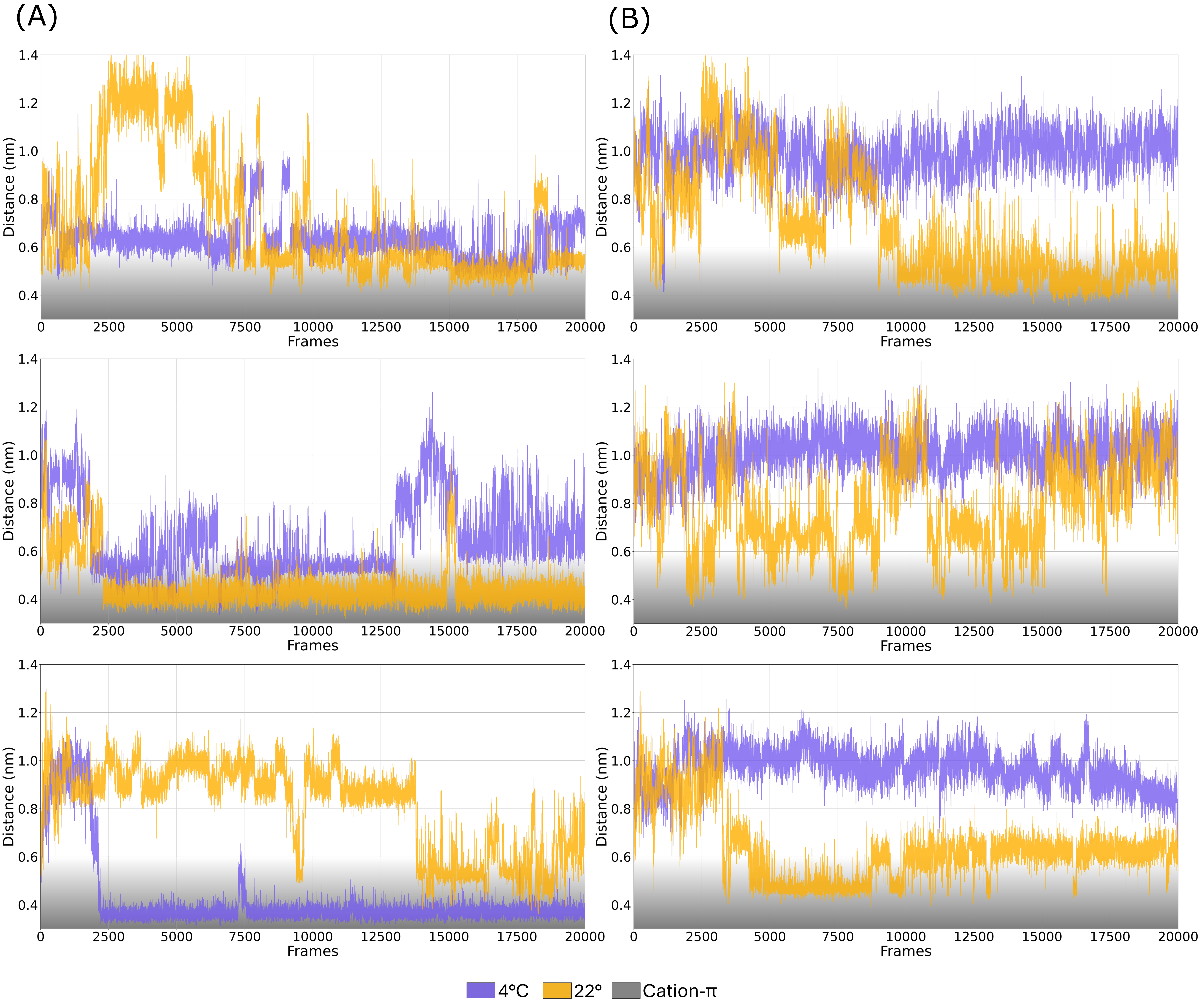

(A)
(B)
Distance (nm)
Frames
4°C
22°
Cation-π

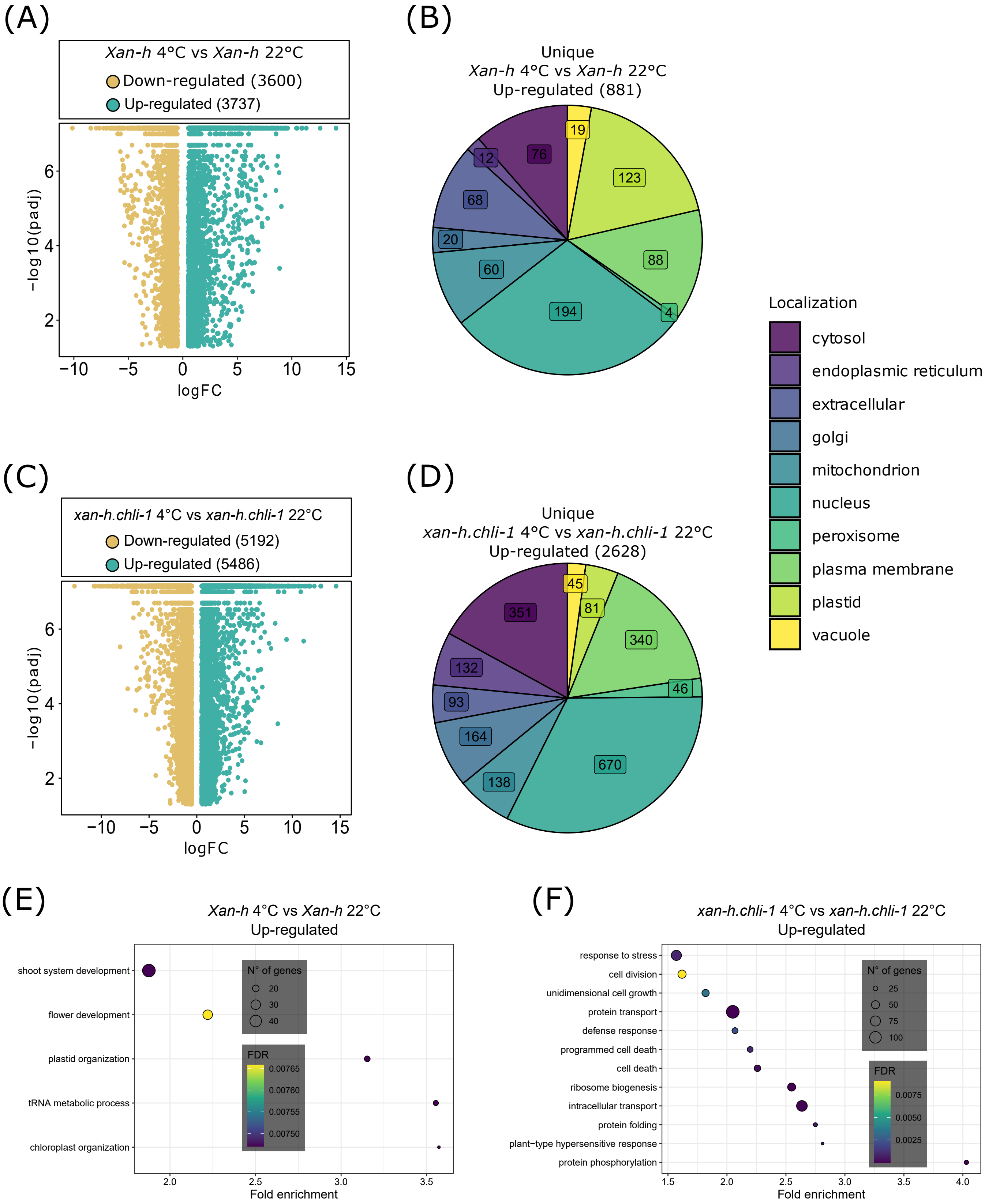
(A)
Xan-h 4°C vs Xan-h 22°C
Down-regulated (3600)
Up-regulated (3737)
−log10(padj)
logFC
(B)
Unique
Xan-h 4°C vs Xan-h 22°C
Up-regulated (881)
19
76
12
123
68
20
88
60
194
4
Localization
cytosol
endoplasmic reticulum
extracellular
golgi
mitochondrion
nucleus
peroxisome
plasma membrane
plastid
vacuole
(C)
xan-h.chli-1 4°C vs xan-h.chli-1 22°C
Down-regulated (5192)
Up-regulated (5486)
−log10(padj)
logFC
(D)
Unique
xan-h.chli-1 4°C vs xan-h.chli-1 22°C
Up-regulated (2628)
45
351
81
340
132
46
93
164
670
138
(E)
Xan-h 4°C vs Xan-h 22°C
Up-regulated
shoot system development
flower development
plastid organization
tRNA metabolic process
chloroplast organization
N° of genes
FDR
Fold enrichment
(F)
xan-h.chli-1 4°C vs xan-h.chli-1 22°C
Up-regulated
response to stress
cell division
unidimensional cell growth
protein transport
defense response
programmed cell death
cell death
ribosome biogenesis
intracellular transport
protein folding
plant−type hypersensitive response
protein phosphorylation
N° of genes
FDR
Fold enrichment
